# Flow cytometric profiling of water buffalo milk EVs: how lactation stage drives phenotypic evolution and macrophage modulation

**DOI:** 10.3389/fvets.2026.1922591

**Published:** 2026-10-06

**Authors:** Barbara Canonico, Mariele Montanari, Ludovica Di Fabrizio, Giulia Franzoni, Giovanna De Matteis, Katia Cappelli, Francesco Grandoni, Maria Carmela Scatà, Daniele Lopez, Samanta Mecocci, Michela Battistelli, Tania Vanzolini, Giovanna Cappelli, Anna Donniacuo, Orlando Paciello, Caterina Ciacci, Stefano Papa, Michele Guescini, Paola Lanuti, Alessandra Martucciello

**Affiliations:** 1 Department of Biomolecular Sciences, DISB, University of Urbino Carlo Bo, Urbino, Italy; 2 Istituto Zooprofilattico Sperimentale della Sardegna, Sassari, Italy; 3 Consiglio per la ricerca in agricoltura e l’analisi dell’economia agraria (CREA), Centro Zootecnia e Aquacoltura, CREA, Monterotondo, Italy; 4 Department of Veterinary Medicine, University of Perugia, Perugia, Italy; 5 National Reference Centre for Hygiene and Technology of Breeding and Buffalo Production, Istituto Zooprofilattico Sperimentale del Mezzogiorno, Salerno, Italy; 6 Department of Medicine and Aging Sciences, University “G.D’Annunzio” Chieti-Pescara, Chieti, Italy; 7 Center for Advanced Studies and Technology (CAST), University “G.D’Annunzio” Chieti-Pescara, Chieti, Italy

**Keywords:** EV-cell priming, flow cytometry, lactation curve, milk EVs, monoclonal antibodies, water buffalo

## Abstract

**Introduction:**

Milk is more than a traditional nutrient source; it serves as a crucial carrier for milk-derived extracellular vesicles (mEVs), food-derived nanoparticles with diverse effects connecting animal health and human nutrition to ecological stability. The primary goal of this study was to characterize the phenotype and functional impact of mEVs from water buffalo (*Bubalus bubalis*) across the lactation curve. The researchers aimed to determine how the transition from colostrum (Colo EVs) to early milk (EM-EVs) and mature milk (MM-EVs) alters the vesicles’ physical properties and their ability to modulate the immune response in macrophages.

**Material and methods:**

The study utilized Flow Cytometry (FC) as a core tool, first validating specific antibody clones for use in the buffalo species. The functional impact was tested on both a bovine cell line (BoMac) and primary water buffalo macrophages, focusing on cellular uptake, mitochondrial oxidative stress (ROS) and phagocytic activity.

**Results and conclusion:**

MM-EVs play the most important role in boosting phagocytosis in bubaline macrophages, accompanied by the highest increase in CD14 surface density, whereas Colo-EVs demonstrated the highest degree of internalization and the strongest antioxidant activity. In conclusion, the study demonstrates that mEVs provide a biological bridge during the calf’s development. While Colo-EVs focus on antioxidant protection and systemic signaling, MM-EVs actively enhance the innate immune defense. This provides a mechanistic explanation for why continued consumption of maternal milk beyond the colostral phase is vital for the immunological resilience and survival of buffalo calves. Furthermore, BoMac cells and bubaline macrophages exhibit distinct cytokine profiles, as IL-1α displays an increasing trend that did not reach statistical significance in MM-EV-treated BoMac cells, whereas a non-significant increasing trend is observed in macrophages treated by both Colo EVs and MM-EVs. Ultimately, the conserved nature of tetraspanins and miRNA profiles underscores the broad cross-species translational potential of buffalo mEVs, extending to human applications.

## Graphical abstract

**Figure:**
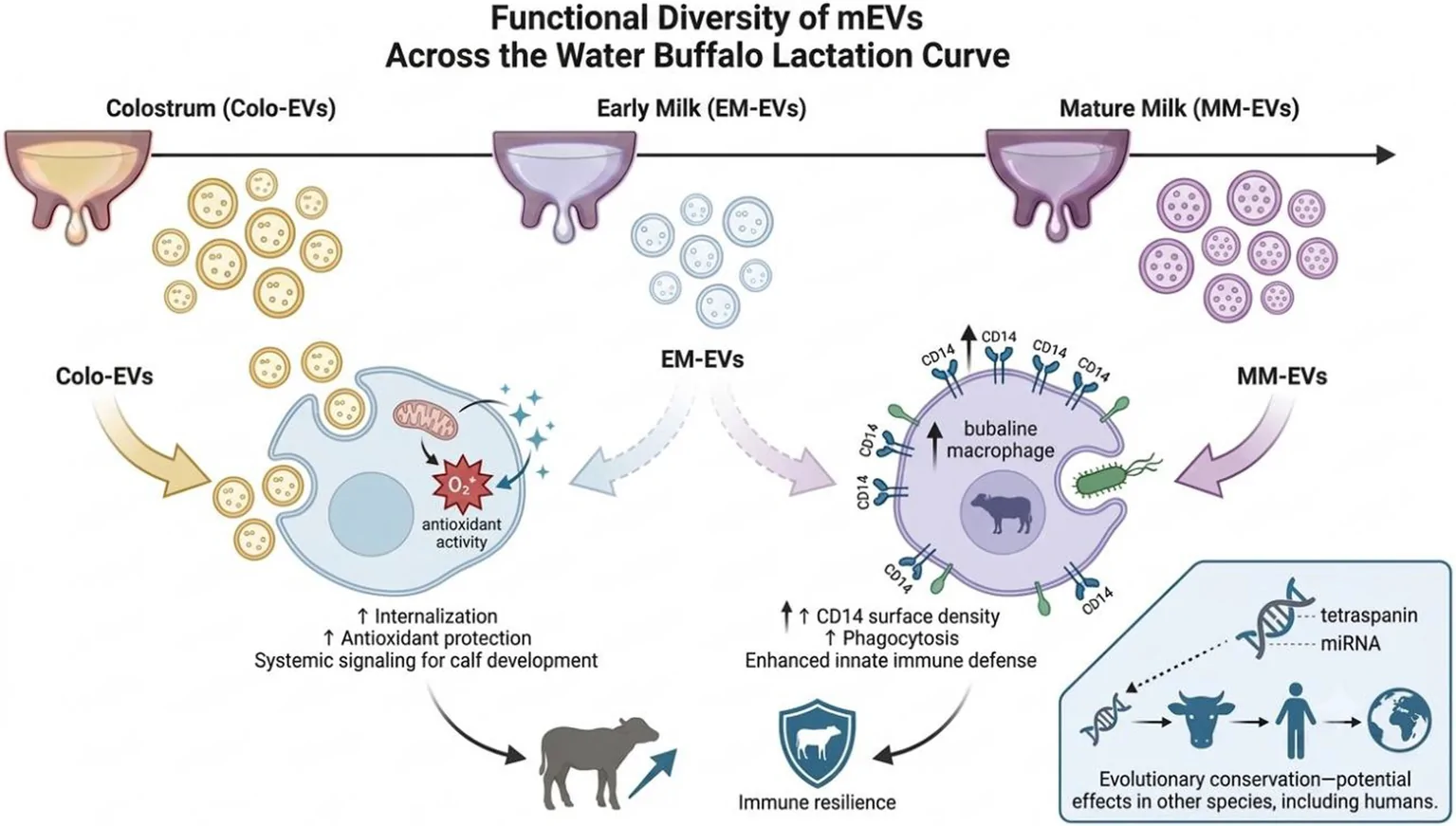

## 1 Introduction

Water buffalo (*Bubalus bubalis*) is an important livestock species in which relatively low artificial selection pressure has allowed the adult to retain a high level of immune resilience. However, at birth, the buffalo calves are in a state of profound hypogammaglobulinemia, with virtually no endogenous antibodies, and therefore depend entirely on the timely ingestion of colostrum for the acquisition of passive immunity. This critical window of vulnerability results in mortality rates that frequently exceed 25% under standard management conditions (1). Once the calf survives the weaning period, a remarkable physiological shift occurs. The adult buffalo develops an extraordinary immunological competence and structural robustness that allows it to thrive in various environments. Differences in EV abundance and cargo represent key distinguishing features between colostrum (Colo) and mature milk (MM). Specifically, EVs are present at higher concentrations in Colo than in milk, acting as crucial carriers of signaling molecules, particularly miRNAs (2, 3). These EVs and their cargo play a vital role in modulating the calf’s immune response and gastrointestinal health, exerting epigenetic regulation that potentially supports long-term physiological resilience and adaptability to environmental and systemic pathogen resistance (4).

mEVs are species-specific in terms of their origin, with different species (e.g., humans, cows, goats) producing unique mEV populations. Indeed, the cargo (including RNA and proteins) within mEVs can also differ between species, although some similarities exist, particularly with closely related species. While mEVs serve to facilitate communication within the same species, their ability to cross biological barriers permits cross-species communication and therapeutic applications (5, 6). The molecular contents of mEVs are not universal; they differ not only on a species basis, but also for breed, diet and health status of animals. Previous research demonstrated that Colo-EVs differ from those in MM, with Colo-EVs generally carrying higher levels of proteins and exhibiting stronger protective effects against intestinal inflammation, while MM-EVs appear more associated with later stages of intestinal barrier function and development. These changes in EV cargo and function during lactation reflect shifts in the mammary gland’s physiological state, impacting intercellular communication and development in the newborn (7, 8). Recently, different researchers (9–11) demonstrated that mEVs can induce immune modulation in cattle by transferring bioactive molecules like proteins and miRNAs. Due to the high evolutionary conservation of miRNA sequences, buffalo mEVs may potentially crosstalk with cells of other species, including humans (4). Moreover, mEVs also act through their surface receptors, using surface proteins for targeted cell interaction and receptor activation: their membrane proteins help them dock and engage specific cells, while the cargo influences downstream effects (12). It is important to cite surface molecules classically employed to detect EVs originating from the Multivesicular Body (MVB) pathway. The International Society for Extracellular Vesicles (ISEV), to improve rigor, reproducibility and transparency in EVs research, provided minimal information for studies of EVs (MISEV2023), including the use of the above-mentioned tetraspanins (mainly CD9, CD63 and CD81) as EV markers. MISEV2023 highlights the importance of reporting pre-analytical variables—such as donor characteristics, sample collection and storage methods—and distinguishing EVs from non-vesicular extracellular particles, which often co-isolate and may confound results (13). In this context, following MISEV guidelines, the current study focuses on flow cytometric identification of mEVs, highlighting differences among Colo-EVs, early milk (EM)-EVs and MM-EVs. EVs of buffalo, even discriminating against the uptake and immunomodulatory effects induced by EVs on both bovine BoMac cells and Water Buffalo macrophages.

The preliminary phase of this study—as is customary in veterinary and animal science—focuses on the evaluation of the efficacy of canonical tetraspanins (CD9 and CD81) specific clones for the successful detection of water buffalo-derived antigens within the milk matrix. Furthermore, using pHrodo Green Zymosan Bioparticles, the study pointed out for the first time that water buffalo MM-EVs play the most important role in boosting phagocytosis in macrophages, accompanied by the highest increase in CD14 surface density. Surprisingly, MM-EVs display moderate internalizing capacity compared to Colo-EVs across both bovine (BoMac) and water buffalo macrophage models. However, MM-EV challenge uniquely promotes a substantial, non-stimulated elevation of pro-inflammatory IL-1α secretion in BoMac cells, highlighting a differential immune engagement between the two macrophage systems.

This study addresses the main questions regarding how water buffalo mEVs influence neonatal immunity and tissue protection. Our aim is to evaluate the distinct functional profiles of MM-EVs and Colo-EVs during inflammation. Understanding these pathways is of high scientific relevance for defining the protective role of maternal milk in early life.

## 2 Materials and methods

### 2.1 Extracellular vesicles obtainment from colostrum, early milk and mature milk whey samples

Briefly, samples of Colo, EM and MM from water buffaloes (*Bubalus Bubalis*) were collected from 11 water buffaloes and processed by Istituto Zooprofilattico Sperimentale del Mezzogiorno. Colostrum samples were collected within the first 6 h post-partum, while milk samples were harvested at two distinct stages of lactation (15 and 60 days). After collection, all samples were maintained under refrigerated conditions, and the preliminary centrifugation steps were completed within 24 h of sampling. Pre-cleared colostrum and milk whey fractions were prepared following the initial steps of the protocol previously described by Mecocci et al. (14), with minor modifications. Briefly, samples were centrifuged twice at 3000 × *g* for 10 min at room temperature using an Eppendorf® Centrifuge 5810R equipped with an F34-6-38 rotor, to remove the superficial fat layer and large cellular debris. The recovered intermediate fraction was then mixed with an equal volume of 0.25 M ethylenediaminetetraacetic acid tetrasodium salt dihydrate (EDTA) pH 7.4, incubated on ice for 15 min, and centrifuged at 10,000 × *g* for 1 h at 4 °C. The resulting pre-cleared colostrum/milk whey fraction was shipped under frozen conditions to the laboratory of University of Urbino and the laboratory of University of Perugia: in both laboratories, EVs isolation was completed.

After thawing on ice, 200 mL of each pre-cleared colostrum or milk whey sample were transferred into ultracentrifuge tubes and centrifuged at 35,000 × *g* for 1 h at 4 °C using a Beckman Coulter Optima L-100 XP ultracentrifuge equipped with a Type45 Ti rotor. The pellet obtained at this stage was discarded, while the supernatant was carefully recovered. This additional pre-clearing step was used to remove residual fat-associated material and casein/protein aggregates. The recovered supernatant (60 mL) was subsequently ultra-centrifuged at 200,000 × *g* for 90 min at 4 °C using the same Type 45 Ti rotor. The resulting EV pellet was resuspended in 2 mL of sterile-filtered PBS/appropriate buffer and stored at −80 °C until downstream analyses. Because the 35,000 × g step may also sediment larger EV populations, the resulting preparation should be regarded as enriched in smaller EVs rather than representative of the total milk EV population (Supplementary Figure S1).

### 2.2 Determination of working dilutions and swarming prevention

To prevent swarming artifacts and ensure reliable single-particle detection in high-density buffalo milk preparations (NTA baseline 10^12^ particles/mL), working sample dilutions were empirically optimized according to MIFlowCyt-EV recommendations. Serial dilutions (1,500 to 1:5.000 reaching also 1:50000) prepared in double-filtered PBS were acquired in the presence of calibrated counting beads Countbright Plus absolute counting beads (Invitrogen™, Thermo Fisher Scientific, Waltham, MA, USA; Cat. #C36995). Operational parameters were maintained at acquisition rates 1.500 events/s, the optimal 1:1000 to 1:5.000 dilution window provided stable back-calculated absolute particle counts while avoiding background noise interference observed at extreme dilutions (Supplementary Figure S2).

### 2.3 Characterization of extracellular vesicles

For the experimental design, both individual animal samples and pooled batches were obtained. Specifically, EV pools for each matrix (Colo-EVs, EM-EVs, and MM-EVs) were obtained by combining equal aliquots from different subjects. Indeed, other EV samples from three individual buffaloes were ultracentrifuged and analyzed alongside the respective pooled samples. For uptake and *in vitro* functional cellular studies, EVs from the three individual animals were utilized and fluorescently labeled using PKH67 (Sigma-Aldrich, St. Louis, MO, USA). As a pre-analytical quality control (QC) step to ensure consistent fluorophore stoichiometry and prevent artifactual effects (dye micelle formation or massive swarming effects) during flow cytometry acquisition, milk whey preparations were evaluated post-centrifugation, and aliquots showing persistent, massive lipid coalescence were excluded before PKH67 labeling, in accordance with MISEV2023 guidelines for lipid-rich biofluids. Briefly, PKH67 was added to the EV suspensions at a final concentration of 2–4 μM in Diluent C, and the mixture was incubated at room temperature (RT) for 5 min. The staining reaction was subsequently stopped by adding 1% bovine serum albumin (BSA). To remove unbound dye and recover the labeled vesicles, the EV suspension was subjected to a final ultracentrifugation step at 100,000 × *g* for 2 h at 4 °C. The resulting fluorescently labeled EV pellets were resuspended and used for downstream uptake tracking.

### 2.4 Transmission electron microscopy observation

The morphological characterization of EVs was performed using TEM, following a standard negative staining protocol. EVs were immediately fixed in 2.5% glutaraldehyde for 30 min, then deposited onto 200-mesh Formvar carbon-coated grids (Agar Scientific Ltd., Stansted, UK) for 10 min to allow adsorption. Subsequently, the grids were stained with a 2% sodium phosphotungstate solution for 1 min. Excess stain was carefully removed using filter paper to prevent artefacts. The samples were then visualized using a Philips CM10 TEM, operated at 80 kV, to assess vesicle morphology, structural integrity and dimension.

### 2.5 Nanoparticle tracking analysis

Nanoparticle tracking analysis was performed using a NanoSight LM10 instrument equipped with an sCMOS camera and a 488-nm blue laser (NanoSight, Amesbury, UK). Before analysis, preliminary dilution tests were performed, and a 1:4000 dilution was selected to obtain an acquisition density of approximately 70–120 particles per frame; the final measurements contained 68.1–118.3 particles per frame and 79.3–124.0 detected centres per frame. For each sample, five videos of approximately 60 s were acquired at 25 frames/s and analyzed using NTA software version 3.4, build 3.4.4. The acquisition settings were camera level 12, shutter 1,200, gain 125, and syringe pump speed 30, while the detection threshold was set to 5. The temperature measured by the instrument during acquisition was 22.5–22.6 °C for Colo-EVs, 23.8–23.9 °C for EM-EVs, and 25.1–25.3 °C for MM-EVs. The corresponding water viscosities automatically applied by the software were 0.939–0.942, 0.911–0.913, and 0.884–0.887 cP, respectively. Particle concentrations were corrected by applying the 4,000-fold dilution factor.

### 2.6 Western blot analysis

Equal protein quantities were separated on 12% SDS-PAGE gel and transferred to 0.2 μm Immuno blot Polyvinylidene Difluoride membranes (PVDF, ThermoFisher Scientific), and successful transfer was confirmed by Ponceau S staining (Sigma-Aldrich). Membranes were blocked with EveryBlot Blocking Buffer (Bio-Rad) for 7 min at RT and incubated overnight at 4 °C with the primary antibody anti-Cytochrome C monoclonal antibody (1:1000; Stressgen, AAM-175). After four washes with 1X TBST (Tris-buffered saline with 0.1% Tween 20), membranes were incubated for 2 h at RT with horseradish peroxidase (HRP)-conjugated secondary antibodies (Bio-Rad). Immunoreactive bands were detected using Clarity Western ECL Substrate (Bio-Rad) and imaged on a Chemi-Doc system (Bio-Rad).

### 2.7 Flow cytometry

Within the MISEV guidelines, we pay attention to the specific MIFlowCyt-EV framework. For FC tests, the initial assessment aimed to establish an efficient screening protocol by identifying reliable tetraspanin markers for buffalo-specific antigens, as reported in the subsequent paragraph. This step is a critical prerequisite in veterinary research to ensure accurate vesicle phenotyping and to account for potential inter-species cross-reactivity. Milk samples were prepared for analysis as described earlier (15). Briefly, to avoid swarming effects, samples were thawed on ice and diluted to 1.500 before analysis by FC, taking into consideration the reports from NTA analyses.

To perform EV characterization, the milk EVs were stained using a combination of the lipophilic dye Labeled Cell Detector (LCD; BD Biosciences, Franklin Lakes, NJ, USA; Custom Kit, Cat. #626267) and anti-tetraspanin monoclonal antibodies: anti-CD9 FITC (clone MM2/57; Bio-Rad Laboratories, Hercules, CA, USA; Cat. #MCA469F), anti-CD63 Alexa Fluor® 488 (clone H5C6; BioLegend, San Diego, CA, USA; Cat. #353016), anti-CD81 PE (clone M38; ExoStep Kit, Immunostep S. L., Salamanca, Spain; distributed by Prodotti Gianni S.r.l., Milan, Italy; Cat. #ExoS-25-CD81). Samples were incubated with 5 μL of each antibody for 60 min at room temperature (RT) in the dark. Following incubation, samples were diluted with 0.22 μm filtered PBS without additional wash steps. For absolute quantification, 10-μL Countbright Plus absolute counting beads (Invitrogen™, Thermo Fisher Scientific, Waltham, MA, USA; Cat. #C36995) were added to each tube. Data acquisition and gating were conducted in strict compliance with MIFlowCyt-EV standards using a FACS Canto II (Becton Dickinson) operated at a low flow rate for 2 min and calibrated according to the manufacturer’s instructions. Single EV events were discriminated from instrument noise using a calibration-defined scatter gate (Rosetta Beads, Exosome Size Range) combined with positive LCD fluorescence to selectively isolate intact membrane-bound mEVs. Specific monoclonal antibody positivity (%) and Mean Fluorescence Intensity (MFI) were defined as relative to Unstained mEV controls (stained with LCD alone) to set the baseline target-channel autofluorescence gate. Buffer alone, sizing beads alone, and ultracentrifuged antibody-only (“mAb-only”) preparations were acquired in parallel to confirm the absence of instrument background noise and free fluorochrome aggregates in the acquisition window. To confirm the presence of intact mEVs per MISEV2023 recommendations, stained samples were incubated with 1% (v/v) Triton X-100 for 1 h at RT prior to acquisition. A reduction in gated event counts post-treatment verified the detergent-sensitive, vesicular nature of the target particles. Post-acquisition analysis was performed using DIVA 6.0 software (Becton Dickinson) as described earlier (15) and as shown in representative plots showing gated sizing bead, enumeration bead and EV populations. A single dilution of the milk sample was used based on our previous study (16).

### 2.8 Evaluation of anti-CD9 efficacy across different species: human, bovine and water buffalo

To verify the cross-reactivity of monoclonal antibodies (mAbs) directed against cluster of differentiation 9 (CD9), whole blood samples from human donors, bovine and water buffalo were analyzed. Two anti-CD9 clones, ML-13 and MM2/57 (Bio-Rad Laboratories, Hercules, CA, USA), were comparatively evaluated (Supplementary Figure S3). 50 μL of whole blood were incubated with 5 μL of each mAb for 20 min at 4 °C in the dark. After incubation, erythrocytes were lysed using 1.0 mL of Tris-buffered ammonium chloride solution (0.87% w/v, pH 7.3) for 10 min. Finally, 4 mL of cold PBS were added, and samples were centrifuged at 300 × g for 5 min. Cell pellets were then resuspended in 150 μL of PBS and acquired using a CytoFLEX flow cytometer (Beckman Coulter, Brea, CA, USA). To confirm the specificity of labelling, criteria previously established by Grandoni et al. (17) were applied.

### 2.9 Water Buffalo blood samples and peripheral blood mononuclear cells separation

Peripheral Blood Mononuclear Cells (PBMCs) were isolated from freshly collected whole blood of healthy buffalo (blood samples were obtained for routine official screening within the mandatory national surveillance program for bovine and bubaline tuberculosis, and the remaining aliquots for 3 individuals were subsequently used for part of this research.). To harvest the monocytes from the peripheral blood, the buffy coats were subjected to a single density gradient centrifugation. A volume of 15–20 mL of buffy coat was layered on top of the Ficoll-PaqueTM Plus solution (GE Healthcare, Little Chalfont, UK) into a 50 mL centrifuge tube for the first density gradient. The sample was centrifuged at 400 × *g* without brake for 30 min at RT, and the white ring of PBMCs, located between the two phases, was collected with a pipette and transferred to a 50 mL centrifuge tube. The tube was filled with PBS 1× (10 mM phosphate buffer, pH 7.3–7.4) up to 40 mL in total, and centrifuged at 300 × *g* for 10 min at RT. The resultant supernatant was discarded, and the pellets were washed with PBS 1x. The pellets were resuspended in RPMI 1640 medium supplemented with 10% (FBS, South America origin, EU Approved; Euroclone S.p.A., Pero, Italy; Cat. #ECS0180L), 2 mM l-glutamine, 100 U/mL penicillin and 100 μg/mL streptomycin and incubated in 5% CO_2_ at 37 °C in six-well tissue culture plates, ensuring uniform baseline conditions across all experimental groups. After 24 h, the non-adherent cells were removed by gentle washing, and the adherent cells left in culture were used for the subsequent assays.

### 2.10 BoMac cell and monocytes/macrophages culture condition

A bovine macrophage cell line (BoMac), kindly provided by Prof. Gaetano Donofrio (Department of Medical-Veterinary Science, University of Parma) and Peripheral blood-derived monocytes (PBMC) were cultured in RPMI 1640 Medium (Sigma- Aldrich, St Louis, MO, USA) supplemented with 10% Heat-Inactivated Fetal Bovine Serum (FBS, South America origin, EU Approved; Euroclone S.p.A., Pero, Italy; Cat. #ECS0180L), 1% l-glutamine (Sigma-Aldrich, St Louis, MO, USA), 1% penicillin/streptomycin (Sigma-Aldrich, St Louis, MO, USA) 1% Amphotericin B and 1% non-essential amino acids at 37 °C in humidified air with 5% CO_2_.

### 2.11 Extracellular vesicle treatments

For the experimental tests, BoMac cells were seeded in 12-well plates at a density of 1 × 10^5^ cells per well and exposed to 2 × 10^4^ EVs/cell for all three types of vesicles used (Colo, EM, and MM) and incubated for 4 h and 24 h. For Ctrl, cells were incubated with culture medium only. This EV-to-cell ratio was selected based on established literature demonstrating that functional cellular responses, transcriptomic changes, and internalization kinetics are highly sensitive to EV concentration. Specifically, an intermediate dosage of 2 × 10^4^ EVs/cell falls within the optimized, biologically relevant window that ensures robust functional signal readouts and accurate tracking of uptake dynamics without inducing cell saturation or cytotoxic artifacts (18–20).

### 2.12 Extracellular vesicle cellular uptake

To quantitatively evaluate the cellular uptake of EVs, BoMac cells and PBMCs were incubated with PKH67-labelled EVs. Specifically, EVs were isolated from 3 different lactation stages (colostrum, early milk (EM), and mature milk (MM)) collected from 3 different individual animals and labeled using the PKH67 Fluorescent Cell Linker Kit. The recipient cells (BoMac and PBMCs) were then exposed to a concentration of 2 × 10^4^ PKH67-labelled EVs per cell. Incubations were performed for 4 h and 24 h at 37 °C in a humidified atmosphere with 5% CO_2_. Following the incubation periods, the dynamics of EV internalization were quantitatively analyzed by FC, while the intracellular localization was assessed using confocal microscopy (CM). Flow cytometry data were analyzed using FACSDiva software. To determine fluorescence intensity changes, autofluorescence values obtained from unstained control samples were subtracted from all stained experimental conditions. Relative fluorescence values (fold increase) were calculated by normalizing the specific Mean Fluorescence Intensity of treated groups to that of the untreated control (CTRL), set as 1.0.

### 2.13 Re-adhesion test and cell count

Following initial incubation, cells were harvested and thoroughly washed to eliminate non-internalized EVs. Cell viability was determined via trypan blue exclusion using a Bürker chamber. To standardize downstream functional readouts, 50,000 viable cells per condition were re-seeded for a 24 h re-adhesion phase in complete culture medium supplemented with exosome-depleted Fetal Bovine Serum (Capricorn Scientific, Ebsdorfergrund, Germany; distributed by Prodotti Gianni, Milan, Italy) to prevent confounding effects from endogenous bovine EVs. Absolute cell counts were subsequently determined by FC using counting beads. Concurrently, intracellular EV retention and persistence were quantified by FC measurement of residual EV-derived PKH67 fluorescence. To exclude artifacts resulting from unbound fluorophore aggregates or free dye carryover, a dye-only control supernatant, obtained from identical staining and wash protocols in the absence of EVs, was acquired concurrently. This wash control was tested both at an equivalent volume ratio and concentrated 100 -fold to validate specific vesicular uptake in full adherence to MISEV2023 and MIFlowCyt-EV standards.

### 2.14 Fluorescent probes for ROS detection and mitochondrial functions

For mitochondrial features and ROS, Tetramethylrhodamine ethyl ester perchlorate (TMRE; Sigma-Aldrich, St. Louis, Missouri, USA) and MitoSOX Red superoxide indicators (Thermo Fisher Scientific, Waltham, Massachusetts, USA) were used. TMRE is a Mitochondrial Membrane Potential (MMP) specific stain able to selectively enter the mitochondria depending on MMP, producing a red fluorescence. TMRE 40 nM was added to the samples 15 min before the acquisition time. MitoSOX Red is oxidized by mitochondrial superoxides, resulting in bright red fluorescence with an excitation wavelength of 561 nm and emission wavelength of 610 nm. Cells were stained with 5 μM MitoSOX Red in PBS for 10 min.

### 2.15 Phagocytosis assay and CD14 staining

To assess phagocytosis, detached bovine macrophages were incubated in the presence or absence (negative Ctrl) of pHrodo Green Zymosan Bioparticles (P35365; ThermoFisher) for 1 h at 37 °C after 24 h of EVs treatment and with pHrodo Green Zymosan Bioparticles on ice (negative Ctrl for phagocytic activity). Phagocytosis was also studied using buffalo macrophages and FITC-labeled *Escherichia coli* (*E. coli*, ATCC 35218). In brief, *E. coli* bacteria were cultured overnight with agitation at 37 °C in Luria-Bertani (LB) medium, pH 7.4. Following visual confirmation of growth, the inoculum was heat-killed by boiling for 20 min, washed twice by centrifugation (4000 rpm, 10 min) and resuspended in phosphate-buffered saline (PBS; NaCl 137 mM, KCl 2.7 mM, Na_2_HPO_4_ 10 mM, and KH2PO4 1.8 mM, pH 7.4). Heat-killed *E. coli* were used instead of live bacteria to prevent bacterial proliferation, isolate baseline phagocytic capacity from active virulence mechanisms, and preserve fluorescence intensity for accurate assay quantification. The bacterial pellet was then resuspended in PBS containing fluorescein isothiocyanate (FITC; Sigma-Aldrich, cat. no. F1628) to a final concentration of 0.5 mg/mL. The bacterial suspension was incubated for 30 min in the dark to allow labeling, followed by three washes in PBS to remove unbound dye. Finally, bacterial cells were resuspended in PBS, the optical density at 600 nm (OD600) was measured, and the suspension was diluted in RPMI to achieve a multiplicity of infection (MOI) of 10:1 (bacteria:cells). Unlabeled bacteria, processed identically but without FITC, served as a negative control (21).

To evaluate the effect of MM-EVs on bacterial interaction, primary buffalo macrophages were pre-treated with or without mEVs before being challenged with FITC-labeled heat-killed *E. coli* (ATCC 35218). Untreated macrophages incubated under identical conditions served as controls. Following incubation, cells were washed to remove non-associated bacteria, detached, and collected by centrifugation for downstream quantitative and spatial analyses. Macrophages were identified using anti-human-CD14 APC antibody (clone M5E2, BD), which has been previously reported to cross-react with buffalo samples. Overall bacteria-cell association was evaluated by quantifying the FITC Mean Fluorescence Intensity (MFI) of the macrophage population via FC. To differentiate surface-bound bacteria from internalized ones, FC MFI quantitative data were complemented with parallel CM image analysis.

### 2.16 Confocal microscopy and quantitative image colocalization analysis

To validate FC population dynamics and investigate the spatial, sub-cellular compartmentalization of extracellular vesicle (EV) uptake, organelle status, and host-pathogen interactions, confocal laser scanning microscopy (CLSM) was performed. For long-term EV uptake and organelle monitoring (24 h), BoMac cells were seeded on 35 mm N-treated glass-bottom dishes (20 mm glass diameter; VWR International, Radnor, PA, USA) at a density of 1 × 105 cells/dish and allowed to adhere overnight. Cells were then treated with EVs for 24 h under adherent conditions. Live cells were subsequently stained with 40 nM TMRE (Sigma-Aldrich) for 20 min at 37 °C to assess mitochondrial membrane potential (MMP), and 100 nM LTDR (Thermo Fisher Scientific) for 20 min at 37 °C to label acidic lysosomal compartments, strictly adhering to the fluorophore incubation protocols optimized for FC assays. For short-term kinetic evaluations (4 h) and direct cross-methodological alignment with FC, BoMac cells were treated with EVs for 4 h under adherent conditions, after which they were detached following the same cell-harvesting procedure used for FC acquisitions. Staining with TMRE (40 nM) and LTDR (100 nM) was performed on detached cell suspensions prior to immediate live-cell confocal imaging in glass-bottom dishes. To evaluate bacterial interaction and discriminate surface-bound from internalized bacteria, PBMCs/macrophages were incubated with FITC-labeled, heat-killed *E. coli* at a cell-to-bacteria ratio of 1:10 for 1 h at 37 °C following EV treatment. Following incubation, samples were thoroughly washed with PBS to remove unbound bacteria and non-associated signals and immediately processed for live-cell confocal acquisition. Images were acquired using a Leica TCS SP5 II confocal microscope (Leica Microsystems, Wetzlar, Germany) equipped with 488 nm, 543 nm, and 633 nm laser lines and a 63×/1.40 NA oil immersion objective. To completely eliminate spectral cross-talk and fluorophore bleed-through, fluorophores were excited with the appropriate laser lines and acquired in sequential scanning mode with custom-tailored spectral detection windows for each dye. Quantitative colocalization analysis was performed on single optical slices and Z-projections using ImageJ/Fiji software (NIH, Bethesda, MD, USA) equipped with the Just another Colocalization Plugin (JaCoP). Spatial correlation between fluorescent signals was quantified by calculating Pearson’s correlation coefficient (*r*) and Manders’ overlap coefficients (M1 and M2). Background thresholding was objectively determined using Costes’ automatic method to avoid user bias. These spatial confocal metrics directly complemented the high-throughput population statistics (Mean Fluorescence Intensity, MFI) obtained via FC.

### 2.17 Lysosomes and autophagic vacuoles monitoring

LysoTracker Deep Red (LTDR) dye (Thermo Fisher Scientific, USA) was used to mark and trace the lysosomes. The LysoTracker is an acidotropic and fluorescent probe which serves for the monitoring of acidic organelles in living cells. The amount of fluorescence obtained by LysoTracker staining is directly proportional to the volume of lysosomes in the cell. LysoTracker 100 nM was used to mark the lysosomes and after 30 min of incubation, the red lysosomal fluorescence was detected by FC. To study the autophagic machinery, the cells were incubated with 50 μM monodansylcadaverine MDC (Sigma-Aldrich, St Louis, MO, USA) to evaluate the autophagic-like vacuoles.

### 2.18 Evaluation of cytokine values in culture supernatants

BoMac cells (*n* = 3 independent biological replicates) and primary water buffalo monocyte-derived macrophages (*n* = 2 independent biological replicates, derived from three healthy animals enrolled in the study) were left untreated (Ctrl) or stimulated with EVs derived from Colo, EM, and MM. At 24 h post-treatment, culture supernatants were harvested, centrifuged at 2,000 g for 5 min to eliminate cellular debris, and stored at −80 °C until analysis. Levels of IL-1α, IL-6, IL-10 and MCP-1 were monitored using the Bovine Cytokine/Chemokine Magnetic Bead Panel Multiplex assay (Merck Millipore, Darmstadt, Germany) and a Bioplex MAGPIX Multiplex Reader (Bio-Rad, Hercules, CA, USA), according to the manufacturers’ instructions (22). All samples were tested in duplicates (2 technical replicates).

### 2.19 Statistical analysis

Data are presented as mean ± standard deviation (SD). All experimental assays, including cytokine quantifications in culture supernatants, were performed in technical duplicates for each sample. To ensure biological reproducibility, 3 independent biological replicates (*n* = 3) were analyzed for BoMac cell experiments. For primary water buffalo monocyte-derived macrophages, although blood was collected from three healthy animals, supernatants from two biological replicates (*n* = 2) were successfully processed and analyzed. Statistical analyses were performed using GraphPad Prism version 9.0.0 (GraphPad Software, San Diego, CA, USA). Data normality was systematically evaluated using the Shapiro–Wilk test. For normally distributed data, two-group comparisons were performed using an unpaired Student’s t-test, whereas multiple group comparisons were analyzed via one-way or two-way Analysis of Variance (ANOVA) followed by Tukey’s post-hoc test. For non-normally distributed data, non-parametric equivalents were applied: the Mann–Whitney U test for two-group comparisons and the Kruskal–Wallis test followed by Dunn’s post-hoc test for multiple group comparisons. Statistical significance was defined as *p* < 0.05 (*p < 0.05, **p < 0.01, ***p < 0.001, ****p < 0.0001).

## 3 Results

### 3.1 Evaluation of cross-reactive tetraspanin reagents for flow cytometric characterization of water buffalo milk extracellular vesicles

To assess tetraspanin expression in compliance with MISEV guidelines (23), anti-human antibody clones previously employed for EV characterization in other mammalian species were evaluated for their cross-reactivity with water buffalo antigens (24, 25). Different labelling were performed and revealed distinct shifts in their fluorescence channels. Notably, CD81 labeling yielded robust fluorescence shifts and expected positive frequencies, consistent with several previous researches (26, 27), confirming the suitability of the selected antibodies and validating our methodology for buffalo mEV analysis. Although the anti-human CD63 clone H5C6 showed detectable staining in buffalo mEVs, the resulting signal lacked the reproducibility required for reliable flow cytometric interpretation. Consequently, this antibody was excluded from subsequent analyses. Indeed, water buffaloes do not always exhibit full sequence homology with bovine counterparts or identical cross-reactivity with human-specific reagents, highlighting the importance of validating these clones.

The human-specific CD9 antibody clone MM2/57 was previously reported as cross-reactive with bovine mEVs. Prior research (28) demonstrates that this clone successfully detects the CD9 protein on bovine cells and their derived EVs. Regarding CD9, the clone ML-13, tested on human, bovine and water buffalo blood samples, showed no detectable cross-reactivity with either bovine or buffalo leukocytes (Supplementary Figure S3) and failed to label buffalo mEVs (Figure 1).

**Figure 1:**
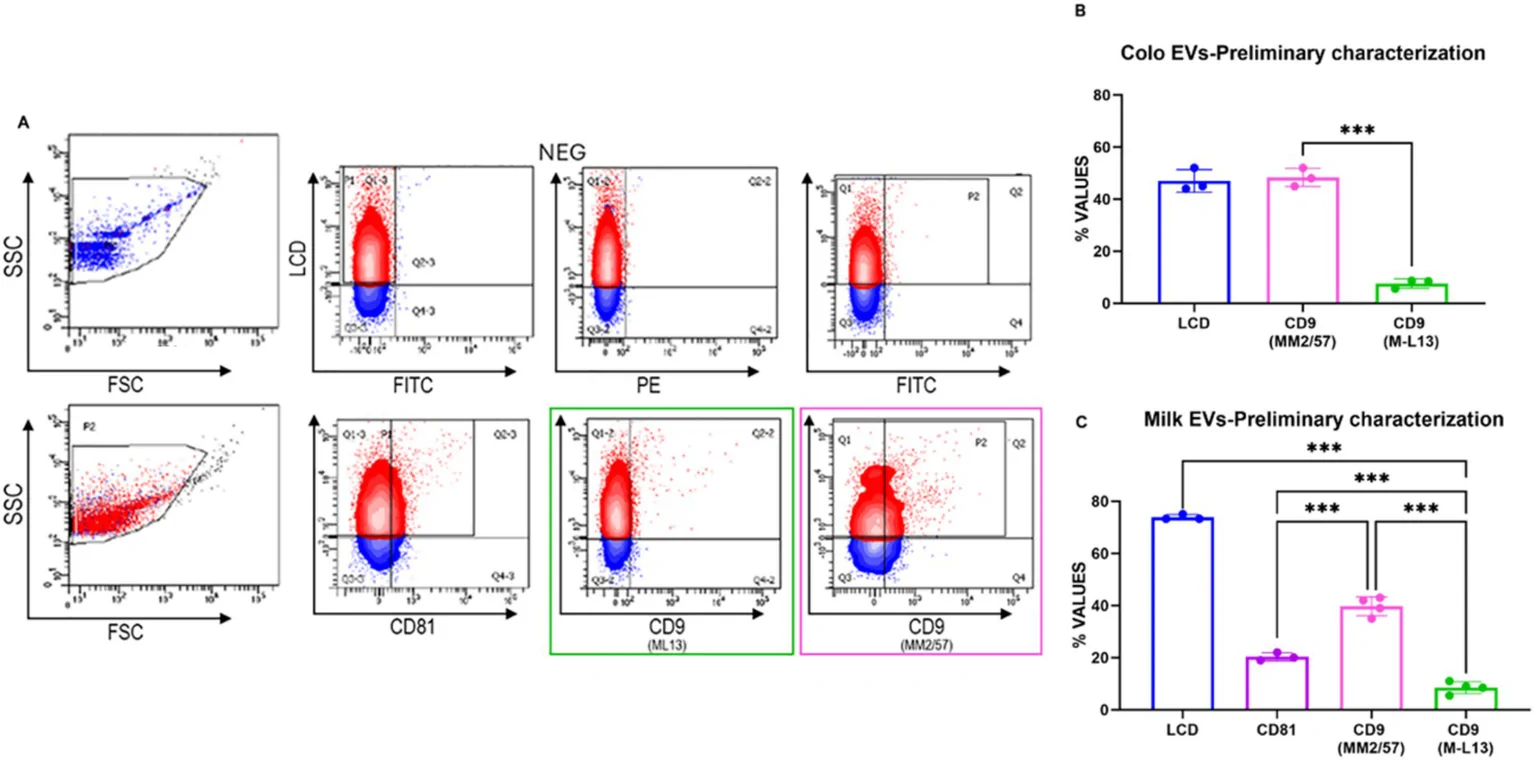
Preliminary FC characterization of EVs from buffalo colostrum and milk whey. **(A)** Gating strategy and FC profiles. Size calibration using Rosetta Beads (SSC vs. FSC dot plot and blue gate). Representative contour plots of EV analysis showing gating for the negative control (NEG; stained with LCD but unlabeled for monoclonal antibodies), the CD81 marker, and a direct comparison for CD9 between the non-reactive M-L13 clone (green box) and the cross-reactive MM2/57 clone (pink box). **(B,C)** Statistical histograms of the preliminary characterization of Colo-EVs **(B)** and Milk EVs **(C)**, expressed as percentages for LCD, CD81, and the two different CD9 clones. The graphs highlight the significantly higher labeling efficiency of the MM2/57 clone compared to the M-L13 clone (*p < 0.05, **p < 0.01, ***p < 0.001).

To overcome the limited fluorescence shift observed with clone M-L13, the alternative anti-human clone MM2/57 (MCA469GA, Bio-Rad; link) was cross-validated using human, bovine, and water buffalo blood samples. Although clone MM2/57 exhibited a lower staining efficiency in water buffalo leukocytes compared to human and bovine samples, it successfully recognized the bubaline ortholog. This cross-validation confirmed that the weak signal previously obtained with clone M-L13 resulted from low binding affinity rather than the absence of CD9 expression. Consequently, clone MM2/57 was validated and selected for further analyses, demonstrating a significantly higher detection efficiency in both Colo-EVs and Milk-EVs compared to clone M-L13 (Figures 1B,C).

### 3.2 Colostrum, early milk and mature milk extracellular vesicles exhibit distinct counts and phenotypic dynamics of mEV tetraspanins across lactation

For FC analyses, EV samples were labeled by LCD, anti-CD9 and anti-CD81 clones, as detailed in the Materials and Methods section. This marker panel was selected and reported based on similar mEV studies, where LCD labeling and tetraspanin profile have been validated as reliable tools for EV detection including in bovine milk (29). Following isolation via ultracentrifugation, EVs from selected Colo, EM and MM samples were first subjected to structural and physical characterization by means of NTA, FC and Transmission Electron Microscopy (TEM). Subsequently, these validated EVs were employed in functional conditioning assays (4 and 24 h) using bovine (BoMac) and bubaline macrophages. NTA was used to assess particle size distribution and concentration in representative Colo-, EM-, and MM-EV preparations, as shown in Figure 2A. The mean particle sizes were 175.6 ± 3.2 nm, 217.1 ± 1.8 nm, and 174.0 ± 1.5 nm, respectively. After correction for the 1:4000 dilution, the corresponding particle concentrations were (4.08 ± 0.18) × 10^12^, (2.92 ± 0.17) × 10^12^, and (2.29 ± 0.06) × 10^12^ particles/mL, respectively. Values are reported as mean ± SEM. Indeed, besides the NTA counts and NTA size estimation, we paired Conventional FC absolute counting and sizing by means of beads and Rosetta Software (Figures 2A–C, Supplementary Figure S4), in agreement with MISEV recommendations for EV orthogonal characterization. Therefore, TEM analyses were also applied to Colo-EVs, EM-EVs and MM-EVs samples, showing the EVs morphology with the cup-shaped lipid layer structure (Figure 2D).

**Figure 2:**
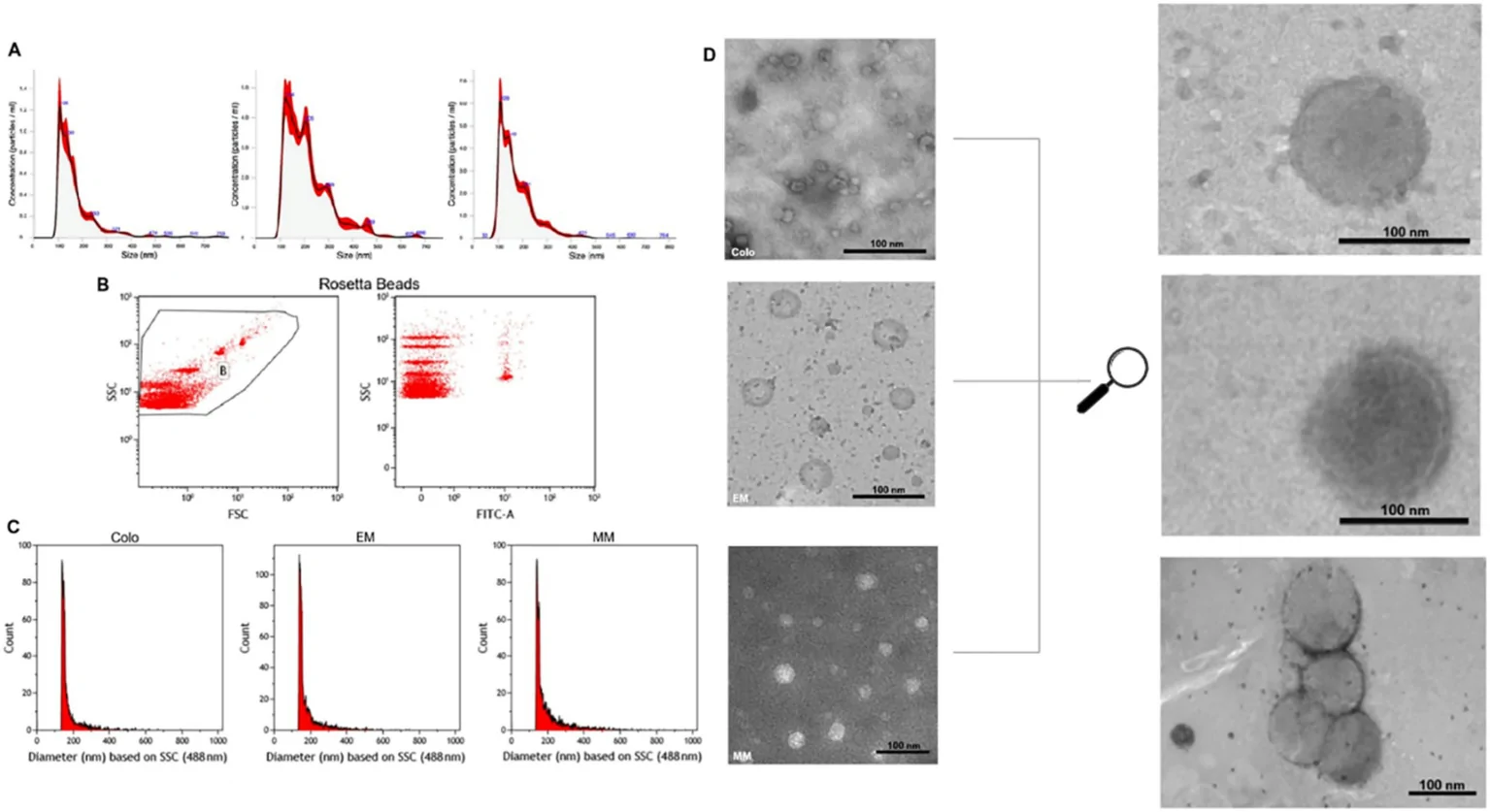
**(A)** Size distribution and concentration profiles across the three different samples by NTA: Colo, EM and MM; **(B)** Representative FC dot plots demonstrating instrument calibration and population gating strategies utilizing standardized Rosetta Beads; **(C)** Dimensional gating encompassing a region of 100–200 nm was drawn on a diameter (nm) histogram (FCS files transformed by the Rosetta Calibration system) representing Colo, EM, and MM-EVs; **(D)** Characterization of EV morphology by TEM (scale bars: 100 nm).

To evaluate the phenotypic dynamics of milk-derived extracellular vesicles (mEVs) across lactation, the expression of CD9 and CD81 was monitored in COLO, EM, and MM mEV preparations (Figure 3). Both tetraspanins were consistently detected across all stages, displaying distinct relative frequencies and surface antigenic densities (MFI ratios relative to colostrum) (Figures 3A–F). Across the lactation curve, a clear temporal modulation was observed: CD9-positive EV frequencies showed a progressive decline from colostrum to mature milk, whereas CD81-positive EVs exhibited an opposite kinetic, increasing significantly in mature milk fractions (Figures 3G–I). Crucially, these expression dynamics were fully consistent and highly reproducible when comparing pooled bulk preparations to individual biological replicates (*n* = 3). Assessing individual animals not only confirmed the overall biological trend observed in bulk samples but also significantly strengthened the statistical confidence (*p* < 0.001) of key temporal shifts in marker frequencies and tetraspanin density. To ensure strict compliance with MIFlowCyt-EV and MISEV2023 reporting standards, fluorescence triggering and sub-micron event gating were validated through a control matrix (Figures 3J,K): Size Calibration: Sub-micron gates were established using standardized Rosetta Beads to accurately delineate the EV acquisition region (Figures 3J, first panel; Figures 3J, first panel). Fluorophore & Carryover Controls (mAb-only): Acquisition of anti-CD9-FITC and CD81-PE alone in PBS confirmed the absence of antibody precipitation or fluorophore aggregates within the sub-micron gate (Figures 3J, second panel; Figures 3K, second panel). Primary Membrane Discrimination (LCD Co-staining): Dual-labeling with a Cationic Lipophilic Dye (LCD) guaranteed that CD9 positivity was restricted exclusively to true lipid-bound particles, successfully eliminating non-vesicular background noise (Figures 3J, fourth panel; Figures 3K, fourth panel). Detergent Sensitivity (Triton X-100): Addition of Triton X-100 resulted in the almost complete dissolution of gated events, confirming that detected signals originated strictly from intact, membrane-enclosed vesicles rather than insoluble protein aggregates (Figures 3J, fifth panel). Importantly, comparative analysis revealed fully consistent expression dynamics and biological trends when evaluating both pooled bulk preparations and individual animal samples.

**Figure 3:**
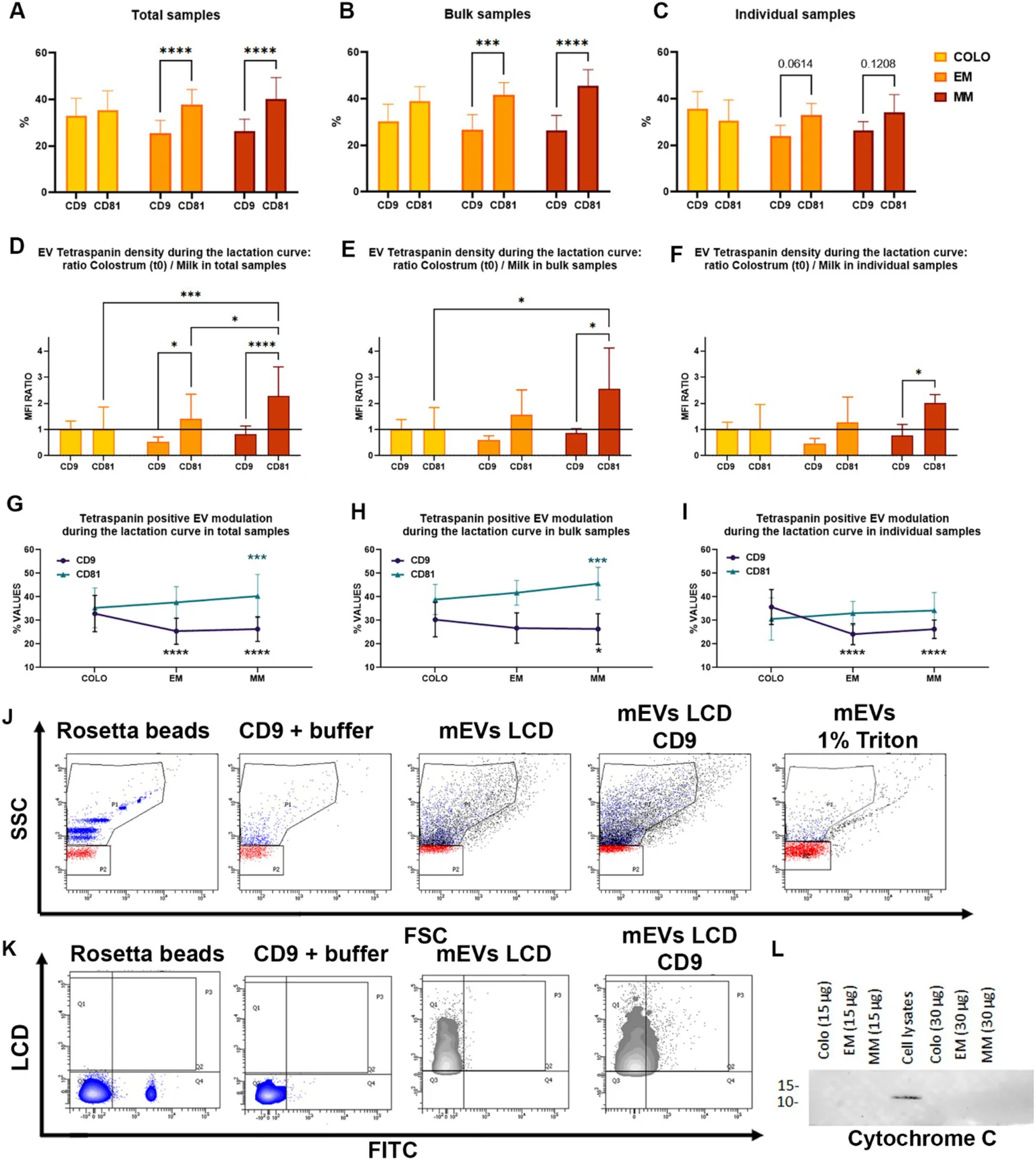
Flow cytometric profiling of tetraspanin expression and density on mEVs across lactation and methodological validation. **(A–I)** FC characterization of CD9 and CD81 tetraspanin expression across Colo, EM, and MM. To account for sample handling and biological variability, data are stratified by total samples **(A,D,G)**, bulk/pooled samples (B, E, H), and individual animal samples **(C,F,I)**. **(A–C)** Percentage of CD9 + and CD81^+^ microvesicles (mEVs). CD81^+^ levels were significantly higher than CD9 in EM and MM across total and bulk cohorts, while individual samples showed a similar distribution without reaching statistical significance ( p=0.0614 for EM; p=0.1208 for MM). **(D–F)** Tetraspanin surface density expressed as Mean Fluorescence Intensity (MFI) ratio normalized to olostrum. CD81 surface density significantly increased in MM compared to EM and Colo in total **(D)** and bulk **(E)** samples **(F)**. **(G–I)** Longitudinal modulation dynamics of CD9^+^ and CD81^+^ positive EV proportions across lactation stages (COLO, EM, MM) in total, bulk, and individual cohorts, respectively **(J,K)** Methodological validation controls for single-EV flow cytometry acquisition according to MIFlowCyt-EV guidelines. **(J)** Forward Scatter (FSC) vs. Side Scatter (SSC) dot plots showing size calibration using Rosetta beads, background signal assessment in antibodybuffer control (CD9 + buffer), stained mEVs (mEVs LCD, mEVs LCD + CD9), and signal specificity verification via detergent lysis (mEVs 1% Triton X-100). **(K)** Corresponding dual-fluorescence density/contour plots (LCD vs. FITC) validating specific antibody binding, gating strategy. (L) Purity assessment of m-EVs. Western blot analysis of the non-EV marker Cytochrome c (~12 kDa) in cell lysates and milk EV fractions (Colo, EM, MM) loaded at 15 μg and 30 μg. The presence of Cytochrome c exclusively in cell lysates confirms the absence of cellular contamination in the isolated EV samples.

### 3.3 Dynamics and subcellular impact of milk extracellular vesicles on BoMac cells: robust antioxidant response and lysosomal decline across the lactation curve

Uptake studies were conducted by means of PKH67 labelled EVs collected along the lactation curve and PKH67 green fluorescence was quantified by FC and morphologically investigated by Confocal Microscopy (CM) approach after both 4 and 24 h post administration. Results highlighted a major uptake for Colo-EVs at both time-points, revealing an internalization for EM-EVs and MM-EVs that was halved compared to Colo-EVs, and only slightly perceptible in CM images (Figure 4). Crucially, flow cytometric analysis confirmed that cellular PKH67 fluorescence was strictly attributable to genuine mEV internalization rather than non-specific dye micelle uptake or residual fluorophore carryover. Cells exposed to the EV-free dye wash supernatant, evaluated both at an equivalent dilution ratio and concentrated 100-fold-displayed fluorescence profiles indistinguishable from baseline untreated controls. In contrast, incubation with Colo-EV-PKH67 resulted in a marked shift in cellular fluorescence (Supplementary Figure S5), validating the specificity of EV-mediated uptake.

**Figure 4:**
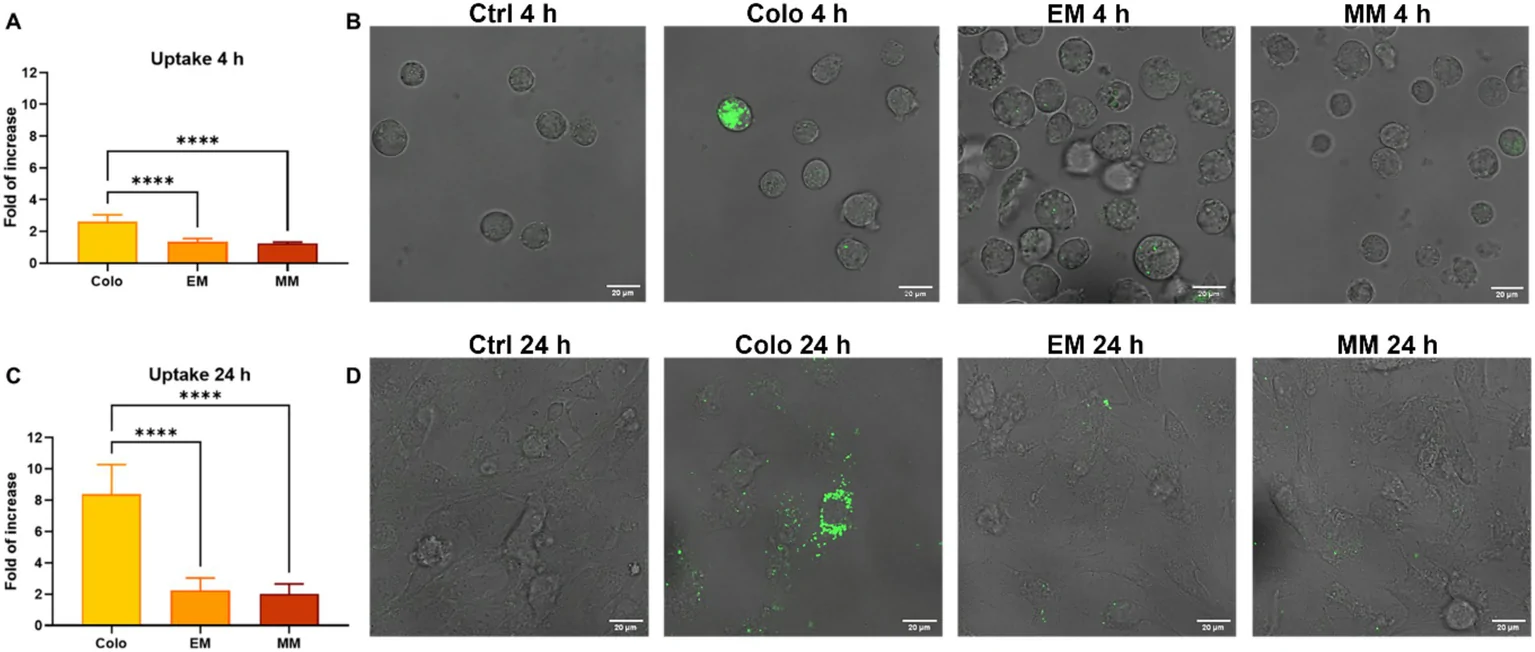
Colo-EVs, EM-EVs and MM-EVs exhibit distinct BoMac uptake and reduce basal mitochondrial ROS. **(A)** Quantitative analysis of cellular uptake after 4 h of incubation in Colo, EM and MM EV-treated cell line. **(B)** Representative fluorescence microscopy images showing the uptake distribution after 4 h, in detached BoMac cells. Scale bar: 20 μm. **(C)** Quantitative analysis of cellular uptake after 24 h of incubation in the same cell line. **(D)** Representative fluorescence microscopy images showing the uptake distribution after 24 h. Scale bar: 20 μm. Data are expressed as mean ± SD of *n* = 3 independent biological replicates. Statistical significance: ****p < 0.0001.

Low 7-AAD positivity (<10%) and normal morphology confirmed that PKH67 uptake was restricted to viable BoMac cells, excluding cytotoxicity-driven artifacts (Supplementary Figure S6). Intriguingly, after 24 h, EM-EVs and MM-EVs approximately doubled their internalization, whereas Colo-EV uptake increased more than 3-fold, significantly higher than EVs collected later during the lactation curve (Figures 4A–D). Overall, these findings demonstrate that, when administering an equal particle number, Colo-EVs exhibit superior internalization in BoMac recipient cells. Regarding oxidative stress, mitochondrial ROS (mtROS) levels were evaluated following EV treatment. A reduction in mtROS was observed after 24 h of EV conditioning, but not at shorter time-points, with Colo-EVs displaying the strongest antioxidant effect. Notably, this reduction in mtROS occurred while mitochondrial membrane potential (MMP) remained unaltered across all conditions (Supplementary Figures S7A–D).

Subcellular effects were further evaluated through the detection of lysosomal and autophagic vacuoles (Figure 5). Quantitative FC analysis revealed a statistically significant contraction of the BoMac lysosomal compartment signal after 24 h of MM-EV administration (Figure 5B), whereas autophagic vacuoles showed no significant variations across conditions (Figure 5D). Confocal microscopy (CM) analysis corroborated these quantitative data (Figure 5 and Supplementary Figure S7E), revealing distinct spatial dynamics upon MM-EV treatment: BoMac cells exhibited a marked centrifugal redistribution of lysosomes, which mobilized from the dense perinuclear cluster toward the cell periphery (Figure 5E). Furthermore, these peripheral lysosomes displayed lower fluorescent intensity and a distinct, more elongated morphology compared to the compact perinuclear distribution observed in control cells.

**Figure 5:**
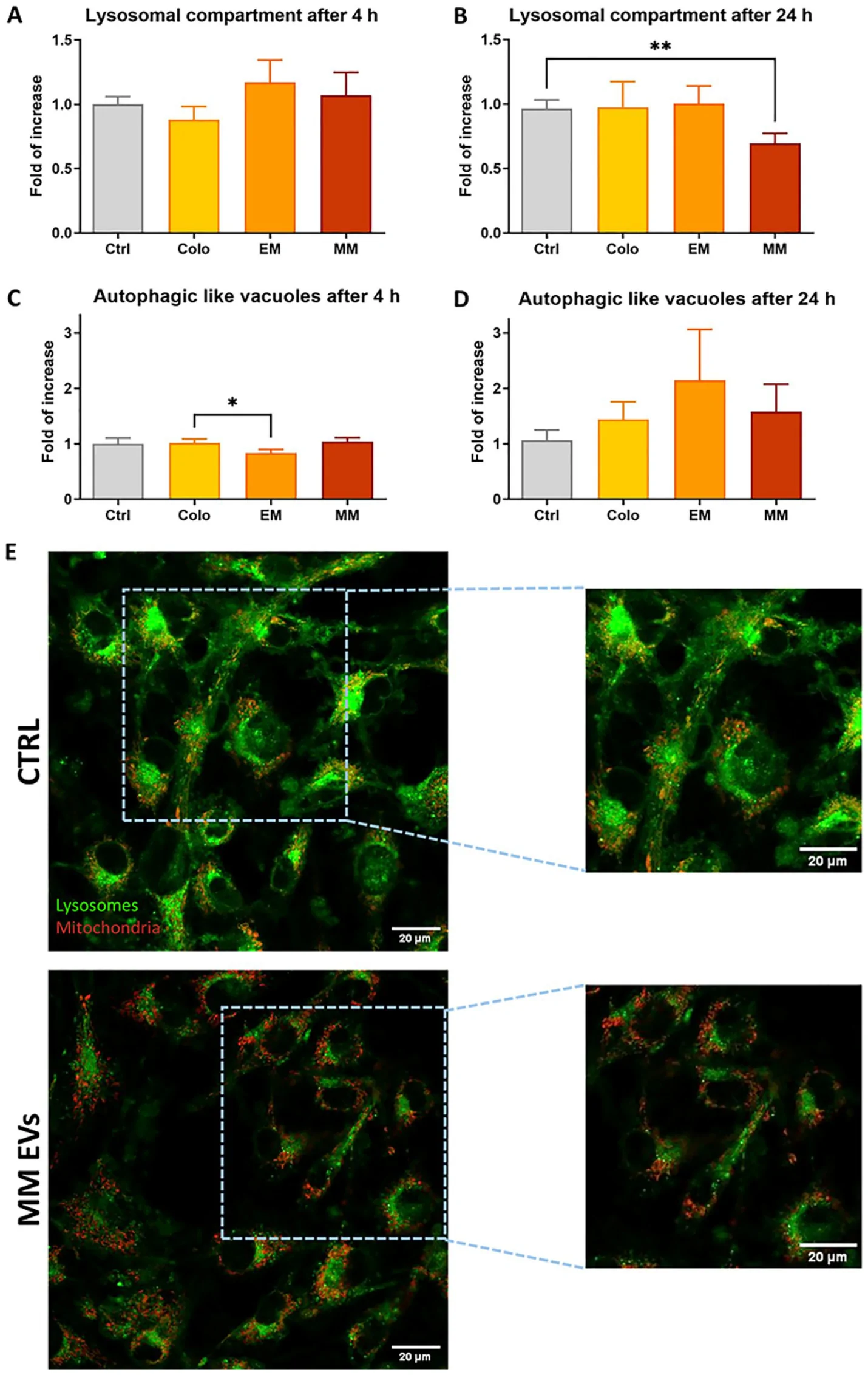
Analysis of the lysosomal compartment and autophagic vacuole formation. **(A,B)** Quantitative assessment of the lysosomal compartment after 4 h **(A)** and 24 h **(B)** of treatment in Colo, EM, and MM-treated cell line. **(C,D)** Quantification of autophagic-like vacuoles after 4 h **(C)** and 24 h **(D)** incubation. Data represents fold of increase relative to Ctrl. Data are expressed as mean ± SD of *n* = 3 independent biological replicates. Statistical significance: *p < 0.05, **p < 0.01. **(E)** Representative fluorescence microscopy images showing the localization of lysosomes (green) and mitochondria (red) in Ctrl cells and MM-EV-treated cells. In Ctrl cells, lysosomes are predominantly clustered in the perinuclear region, whereas in MM-EV-treated cells, they appear more peripherally distributed toward the cell periphery. Higher magnification inserts (right panels) highlight these morphological details within the dashed areas. Scale bars: 20 μm.

### 3.4 The processes of persistence and exit of milk extracellular vesicles in BoMac cells occur without affecting adhesion, proliferation and redox balance

Data highlighted in Figure 6 suggest that Colo-EVs, EM-EVs, and MM-EVs exhibit differential intracellular persistence and fate within BoMac macrophages without compromising host cell viability. Following either 4h or 24h EV exposure, intracellular PKH67 fluorescence retention and cellular counts were evaluated after a subsequent 24h chase period. Quantitative assessment of intracellular signal revealed a significantly lower retention of Colo-EV-derived fluorescence compared to EM-EVs and MM-EVs (Figures 6A–D). To investigate whether this drop in intracellular content corresponded to an outward trafficking route, extracellular PKH67 + particulate events were quantified in the conditioned medium (Figures 6E,F). Notably, a markedly higher count of PKH67 + fluorescent events was detected in the culture supernatant of Colo-EV-conditioned cells (Figures 6E,F). While non-specific lipophilic dye transfer or micellar diffusion represents a recognized analytical constraint in membrane-labeling assays, this possibility is strongly mitigated by our validated dye-only wash control. These data points toward a distinct intracellular processing model for Colo-EVs compared to more mature stages. While MM-EVs appear mainly routed toward the lysosomal pathway for intracellular processing and degradation (Figure 7A) Colo-EVs appear to engage in an alternative processing pathway. This mechanism facilitates the outward release of vesicle-derived membrane components (Figures 6E,F), potentially serving as a mechanism to influence neighboring cells and immune populations (29) while maintaining full cell functions (Supplementary Figure S8).

**Figure 6:**
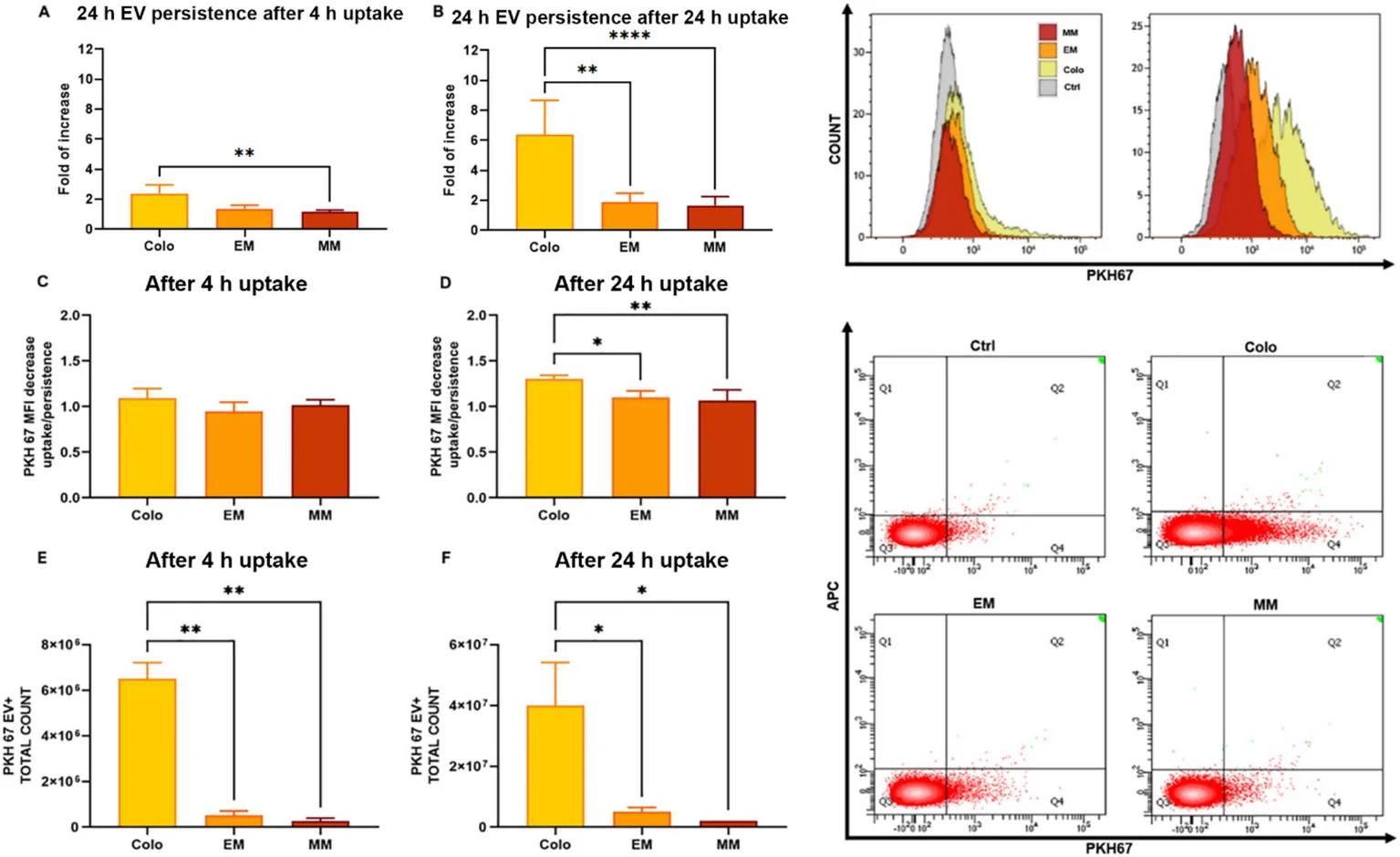
Persistence of EVs in BoMac cells and evaluation of re-release bouts. (A, B) Evaluation of 24 h EV persistence in BoMac cells following an initial 4 h **(A)** and 24 h **(B)** uptake. (C, D) PKH 67 mean fluorescence intensity (MFI) decrease in BoMac cells, calculated as the uptake/persistence ratio after 4 h **(C)** and 24 h **(D)** uptake. **(E,F)** Total count of PKH 67 + EVs in the medium measured after the initial 4 h **(E)** and 24 h **(F)** uptake phases. On the right, representative FC overlay histograms (top) illustrate intracellular PKH67 fluorescence for EV persistence after 4 h and 24 h of uptake. The representative contour plots (bottom) evaluate the presence of re-released EVs in the culture medium from the same cells left adhering overnight. Data are expressed as mean ± SD of *n* = 3 independent biological replicates. Statistical significance: *p < 0.05, **p < 0.01; ****p < 0.0001.

**Figure 7:**
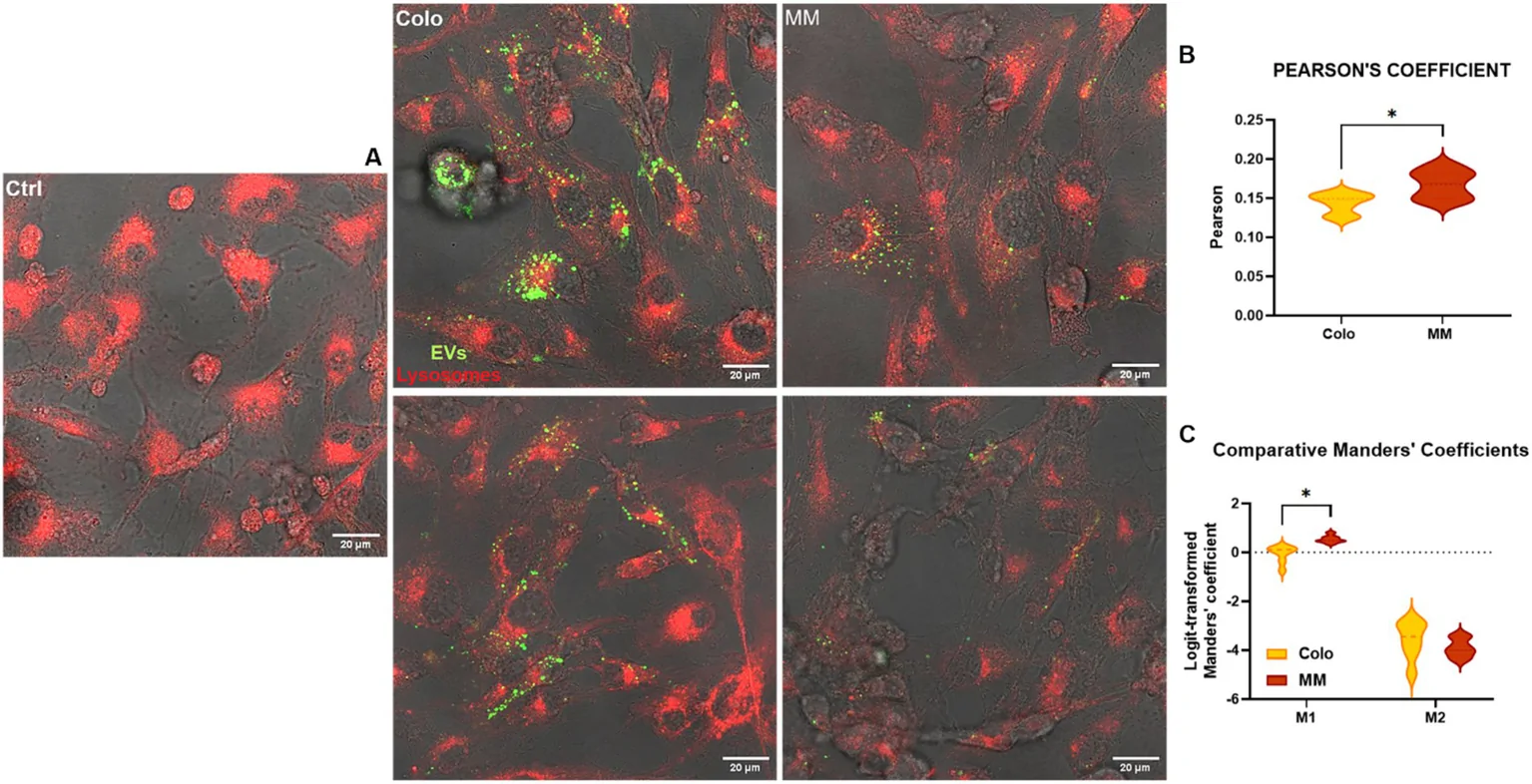
Lysosomes are more successfully reached by MM-EVs than Colo-EVs. **(A)** Representative CM images: Intracellular uptake of green-fluorescent labeled EVs derived from Colo and MM. Scale bars represent 20 μm; **(B)** Pearson’s correlation coefficient analysis: MM-EVs show a significantly higher Pearson’s coefficient compared to Colo-EVs; **(C)** Comparative Manders’ co-localization coefficients: M1 represents the fraction of EV signal overlapping with the cell channel. M2 represents the fraction of cell signal overlapping with EVs.

While more mature mEVs are predominantly routed toward the lysosomal compartment, to be involved in both endosomal escape and lysosomal degradation (Figure 7A), Colo-EVs are also engaged in “exit processes” where macrophages can re-release EV components, in agreement with previous findings (30), to influence neighboring cells and other immune populations (31). Importantly, these dynamics did not compromise host cell viability. No significant decrease was observed in BoMac cell counts 24 h after a 4-h conditioning with Colo-EVs and EM-EVs, with only a mild reduction in the MM-EV group. Furthermore, a full 24-h EV conditioning period showed no negative impact on cell adhesion or proliferation, demonstrating that BoMac cells remain perfectly functional and fully retain their physiological capabilities after interacting with mEVs (Supplementary Figure S8). In particular, a very low intensity correlation was found for both vesicle types, as indicated by the low Pearson’s coefficients (Figure 7B), although the value for MM-EVs was significantly higher than that of Colo-EVs. On the other hand, a marked spatial overlap was observed specifically for MM-EVs within lysosomes, as revealed by the Manders’ coefficient (Figure 7C). Interestingly, analysis of EM-EVs revealed an intermediate profile (Supplementary Figure S9A): while EM-EVs displayed a marked reduction in lysosomal occupancy (M2) compared to Colo-EVs at 24 h, they did not reach the high M1 co-localization efficiency observed for MM-EVs (Supplementary Figures S9B,C). This scenario suggests that lysosomes may vary in their acidification levels and that degradation might have already started, thereby quenching the EV fluorescence while the lysosomal marker remains bright. Consequently, while MM-EVs are efficiently targeted to the lysosomal pathway for degradation, Colo-EVs largely evade this route, remaining available for intercellular re-release.

### 3.5 Water buffalo mature milk extracellular vesicles trigger the strongest phagocytic response and upregulate surface CD14 in bubaline macrophages

While BoMac serves as a useful cell line model, their limited phagocytic ability means they do not fully represent the strong immune response of bovine (or bubaline) macrophages, which can significantly enhance phagocytosis with serum (opsonization). For this reason, only phagocytosis tests conducted on buffalo macrophages are essentially decisive. Bubaline macrophages were obtained from bubaline peripheral samples and maintained in adhesion for 1 week, following a protocol set up on human cells (32). Based on preliminary functional profiles established in BoMac cells, which identified early (Colo) and late (MM) lactation phases as the most representative conditions and given the strictly limited cell yields obtained from primary monocyte isolations, subsequent phagocytic evaluations were strategically focused on Colo-EVs and MM-EVs.

After 24 h of Colo-EVs and MM-EVs administration, phagocytosis tests were performed, highlighting a mild, not significant increase in the phagocytic process in Colo-EV-treated macrophages and, concomitantly, a stronger increase in MMEV-treated macrophages, where CD14 surface density was also significantly increased. These results highlight a stage-specific effect of mEVs, with MM-EVs significantly boosting the scavenge-related phenotype (CD14^+^) and the primary defensive function (phagocytosis) of buffalo macrophages. Since in the same test, labelled EVs cannot be employed (PKH67 and pHrodo exhibit similar fluorescence channel), macrophage uptake was separately evaluated (Supplementary Figure S10), highlighting a slightly not significant minor uptake for MM-EVs in respect to Colo-EVs. The narrower gap observed between Colo-EVs and MM-EVs, compared to the findings in BoMac cells, may be attributed to species-specificity.

Given that only MM-EVs significantly enhanced both CD14 expression and phagocytic activity (Figures 8A–D), this specific fraction was selected for further evaluation in the *E. coli* uptake model. In fact, a test with FITC-labelled heat-killed *E. coli* was set up at 1 h post-administration of unlabeled MM-EVs: such timing is justified as mEVs initiate contact with the intestinal immune environment almost immediately in a calf model (mEVs can bypass the rumen and reach the small intestine within minutes), allowing a rapid interaction with the immune cells of the gut lamina propria.

Of note, FC quantification of FITC fluorescence (Figure 8E) was coupled with CM analysis, a step enabling us to appreciate that the decrease in FITC MFI resulted from a 2.5-fold reduction in *E. coli* adhesion to the macrophage surface (Figure 8F).

**Figure 8:**
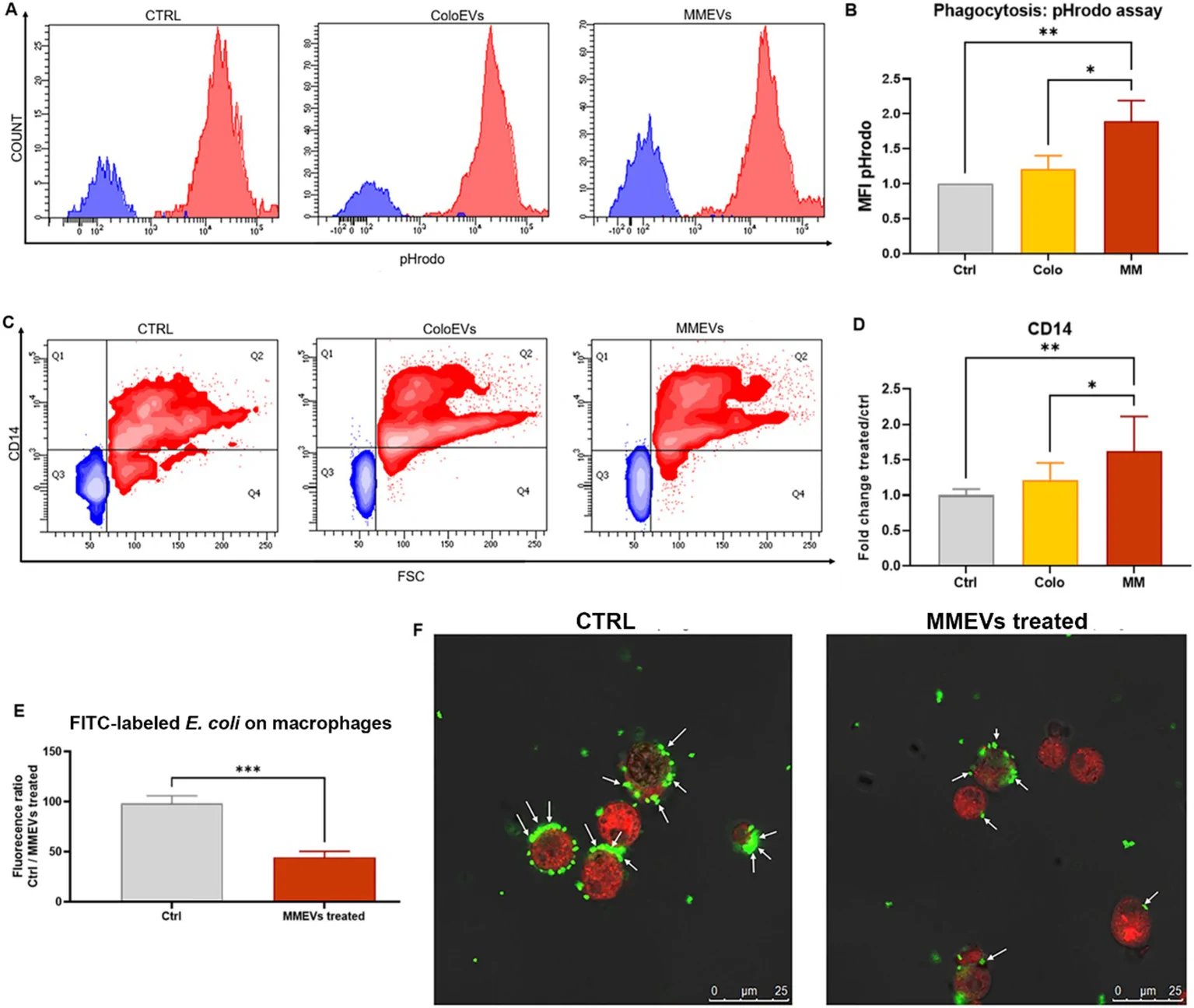
EVs modulate phagocytic response and CD14 expression. **(A)** FC histograms of pHrodo MFI for Ctrl, Colo and MM EV-treated cells (blue: residual lymphocytes, red: macrophages); **(B)** Statistical histogram of phagocytosis quantification obtained by pHrodo assay in macrophages; **(C)** FC contour plot of FSC vs. CD14 of Ctrl, Colo and MM EV treated cells; **(D)** Statistical histogram of CD14 expression quantification in «red» macrophages; **(E)** Statistical histogram representing the comparison of *E. coli* and MMEV-treated *E. coli* adhesion treatments on buffalo PBMCs where we can observe an inhibition of the bacterial adhesion; **(F)** CM images of FITC labelled *E. coli* (green on buffalo PBMCs; red lysosomes).

### 3.6 Dynamics of EV-mediated cytokine modulation across the lactation curve

As presented in Figure 9A, treatment of BoMac cells with MM-EVs induces a relevant increase in IL-1α release, although not significant, whereas the levels of IL-6, IL-10 and MCP-1 in culture supernatants were not affected. The release of IL-1α by BoMac was simultaneous with a reduction in ROS levels, as previously shown. In addition, BoMac did not release any of the tested cytokines in response to stimulation with Colo-EVs or EM-EVs (Figure 9A). Next, we investigated the response of bubaline macrophages to stimulation with Colo-EVs, EM-EVs and MM-EVs.

**Figure 9:**
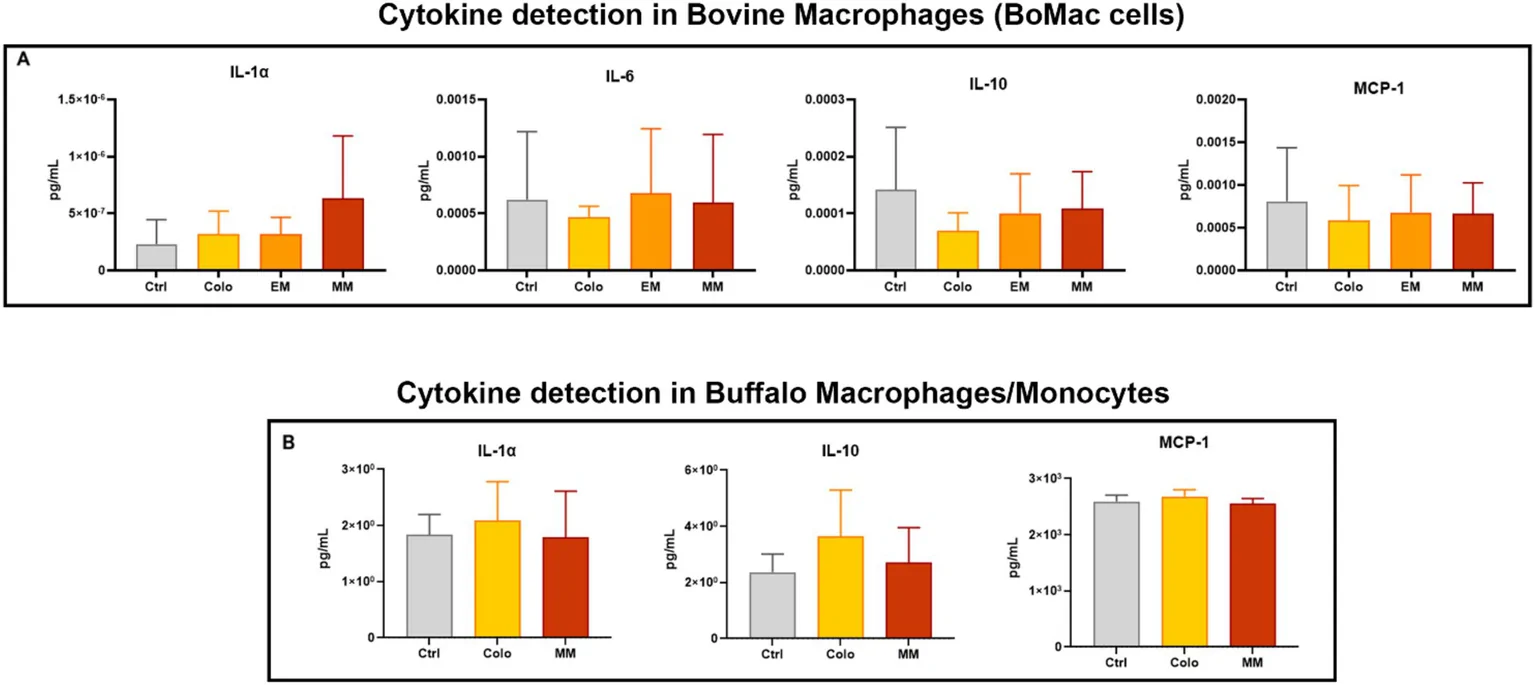
Water buffalo mEVs differently modulate cytokines across the lactation curve with evidence of species-specific impact **(A)** cytokine detection in BoMac cells: Data from 6 biological replicates are presented; **(B)** cytokine detection in water buffalo monocytes/macrophages: Data from 2 diverse blood donors are displayed.

As presented in Figure 9B, data on bubaline macrophages highlights that water buffalo mEVs exert only mild cytokine modulatory effect. Specifically, the treatment with Colo-EVs appears to upregulate the production of the anti-inflammatory cytokine IL-10 compared to the Ctrl (Figure 9B), although not significantly. The levels of IL-10 and MCP-1, remained relatively stable (Figure 9B).

This different behavior between the two cell models may stem from their classification into two distinct species (bovine and buffalo), which, as is well established, share numerous similarities while retaining unique, distinctive characteristics (33).

## 4 Discussion

The present study demonstrates that the immunomodulatory potential of mEVs is stage-specific across the lactation curve, with MM-EVs exerting the most pronounced effects on macrophage activation. In line with MISEV2023 standards, the identification of endosomal-derived mEVs in complex matrices such as milk requires highly validated markers. The selection of anti-tetraspanin antibodies was central to our FC approach: cross-reactivity led to the choice of anti-CD81 clone M38, whereas selecting an anti-CD9 antibody required extensive screening, resulting in the reliable use of clone MM2/57. Utilizing these clones, we established the percentage distribution, and antigenic density of CD9 and CD81 across lactation. Furthermore, anti-tetraspanin MFI labeling highlighted a progressive shift in surface marker expression aligning with human serum *vs*. milk EV dynamics. The temporal shift in tetraspanin surface density observed in our study provides a mechanistic rationale for the inconsistent capture efficiencies frequently reported in literature (26, 34, 35) when analyzing mEVs via bulk bead-based immunoassays. While multiplex bead assays heavily rely on high epitope density and strong avidity for vesicle capture often leading to underestimation or targeting failure when specific markers like CD9 are downregulated across lactation stages, our single-event FC approach seems to resolve these sub-populational kinetic shifts at the single-vesicle level. Although non-vesicular matrix components, such as hydrophobic casein micelles, may exhibit partial resistance to non-ionic detergents like Triton X-100 (35–37). Our dual-staining strategy correctly defined true mEVs as double-positive events LCD+/Tetraspanin+. This gating strategy effectively excluded non-vesicular protein aggregates, as single-stained or non-specifically labeled casein structures lacked specific surface tetraspanin reactivity.

To evaluate functional uptake and downstream signaling, BoMac cells (mainly) and primary water buffalo monocytes/macrophages were employed as recipient models. While BoMac cells provide a standardized bovine model to minimize biological variance, they exhibit lower phagocytic efficiency and lack key receptors such as integrins. Interestingly, Colo-EVs displayed superior internalization in BoMac cells compared to later mEVs, suggesting higher initial surface affinity or evolutionary adaptation for immediate post-natal immune crosstalk. Furthermore, 24 h conditioning with Colo-EVs significantly reduced mitochondrial ROS (mtROS) without altering mitochondrial membrane potential (MMP). This indicates that mtROS attenuation is not driven by mitochondrial collapse, but rather by the delivery of bioactive cargo that stabilizes the electron transport chain, neutralizes free radicals, and enhances respiratory efficiency. Flow cytometric quantification and confocal imaging revealed that MM-EV treatment induces a quantitative contraction and centrifugal redistribution of the lysosomal compartment toward the plasma membrane, alongside a decrease in acidic fluorescence intensity. This peripheral redistribution is indicative of active lysosomal exocytosis, membrane repair, and enhanced bacterial clearance capacity, suggesting that MM-EVs functionally condition macrophages toward a distinct endo-lysosomal and secretory state. Given the inherent limitations of BoMac cells, primary bubaline macrophages were utilized to assess phagocytosis and immune maturation, a model of high relevance for *Bubalus bubalis*, a species characterized by extreme neonatal fragility alongside robust adult immunity [45]. Our data reveals a potential functional division of EVs: while Colo-EVs specialize in antioxidant protection and intracellular transport (bypassing lysosomal degradation to reach distant tissues), MM-EVs excel in enhancing phagocytosis, upregulating CD14, while promoting an upward trend in IL-1α secretion. As an alarmin, IL-1α is reported to recruit neutrophils and drive an M1-like pro-inflammatory phenotype essential for early pathogen defense. Thus, while Colo-EVs provide passive immune protection, MM-EVs seem to deliver essential signals for local innate surveillance and systemic immune maturation as the calf grows, explaining why prolonged maternal milk consumption enhances disease resilience. From an applied perspective, these findings support the formulation of artificial milk replacers enriched with stage-specific mEV fractions. Such bioactive supplementation represents an economically viable strategy to accelerate functional immune development and significantly reduce neonatal mortality in buffalo calves.

## Data Availability

The raw data supporting the conclusions of this article will be made available by the authors, without undue reservation.
