# Supplementary material for: Flow cytometric profiling of water buffalo milk EVs: how lactation stage drives phenotypic evolution and macrophage modulation
Source: Front Vet Sci. 2026 Oct 6;13:1922591. doi: 10.3389/fvets.2026.1922591 (PMC13681838; doi:10.3389/fvets.2026.1922591)
Supplement: Supplementary file 1 [file Supplementary_file_1.PDF]

Supplementary Material of

Flow Cytometric Profiling of Water Buffalo Milk EVs: How Lactation Stage Drives Phenotypic Evolution and Macrophage Modulation

Canonico Barbara[1], Montanari Mariele[1], Di Fabrizio Ludovica[1*], Franzoni Giulia[2], De Matteis Giovanna[3], Cappelli Katia[4], Grandoni Francesco[3], Scatà Maria Carmela[3], Lopez Daniele[1], Mecocci Samanta[4], Battistelli Michela[1], Vanzolini Tania[1], Cappelli Giovanna[5], Donniacuo Anna[5], Paciello Orlando[5], Ciacci Caterina[1], Papa Stefano[1], Guescini Michele[1], Lanuti Paola[6,7], Martucciello Alessandra[5]

[1] Department of Biomolecular Sciences, DISB, University of Urbino Carlo Bo, 61029 Urbino, Italy;

[2] Istituto Zooprofilattico Sperimentale della Sardegna, Sassari, Italy

[3] Consiglio per la ricerca in agricoltura e l'analisi dell'economia agraria (CREA), Centro Zootecnia e Aquacoltura, CREA, 00015 Monterotondo, Italy;

[4] Department of Veterinary Medicine, University of Perugia, 06126 Perugia, Italy;

[5] National Reference Centre for Hygiene and Technology of Breeding and Buffalo Production, Istituto Zooprofilattico Sperimentale del Mezzogiorno, 84131 Salerno, Italy;

[6] Department of Medicine and Aging Sciences, University "G.D'Annunzio", Chieti-Pescara, 66100 Chieti, Italy;

[7] Center for Advanced Studies and Technology (CAST), University "G.D'Annunzio", Chieti-Pescara, 66100 Chieti, Italy.

*** Correspondence:**
Corresponding Author: l.difabrizio1@campus.uniurb.it

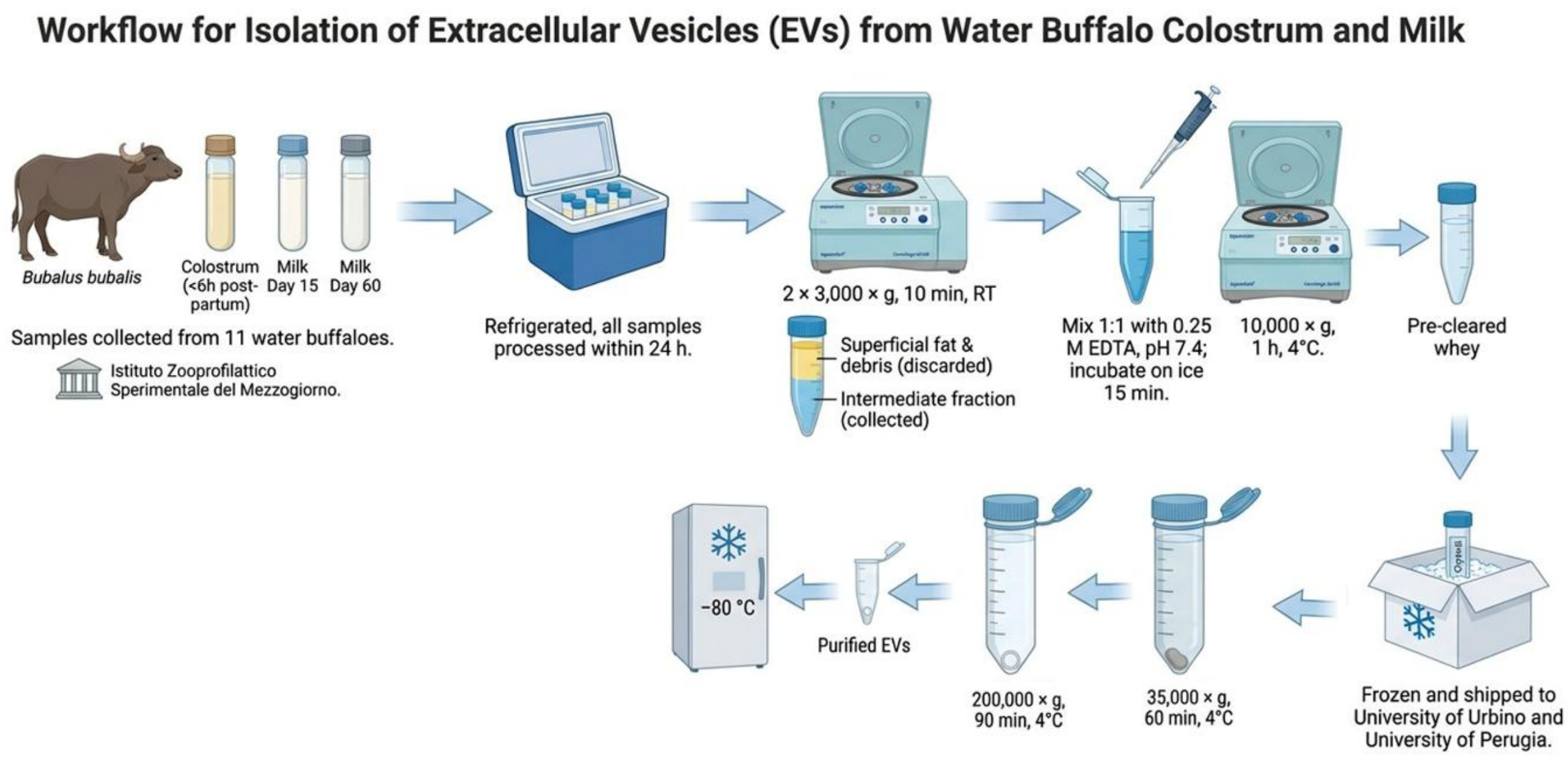

Supplementary Figure 1: Operational Workflow for EV Isolation from Buffalo colostrum and milk.

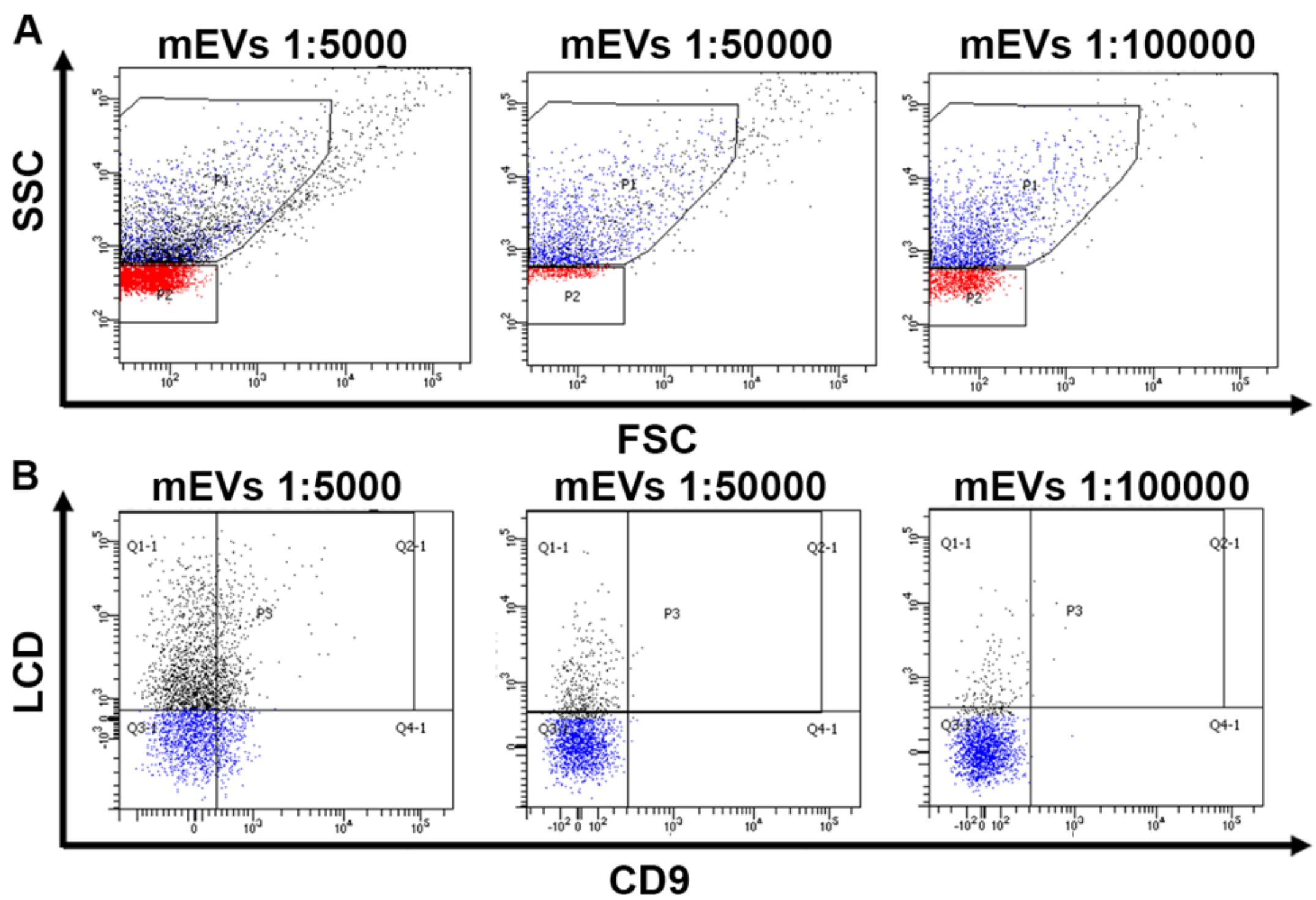

Supplementary Figure 2. Serial dilution analysis to assess single-particle resolution and background interference in microvesicles (mEVs). (A) Representative forward scatter (FSC) versus side scatter (SSC) dot plots of mEVs acquired at serial dilutions (1:5000, 1:50000, and 1:100000). While events within the physical gates (P1 and P2) persist across all dilutions without a proportional

decrease, this physical signal consists primarily of instrument noise and buffer particulates. **(B)** Dual-fluorescence plots (LCD vs. CD9) across the corresponding serial dilutions. Specific staining with the lipophilic membrane marker (LCD) and CD9 confirms that true mEVs are clearly detected at 1:5000 (LCD^+ population). Upon further dilution to 1:50000 and 1:100000, the $LCD^+/CD9^+$ events disappear, demonstrating that the sample becomes over-diluted. Thus, LCD staining serves as a critical control to distinguish intact biological mEVs from non-vesicular background signals present in the scatter gates.

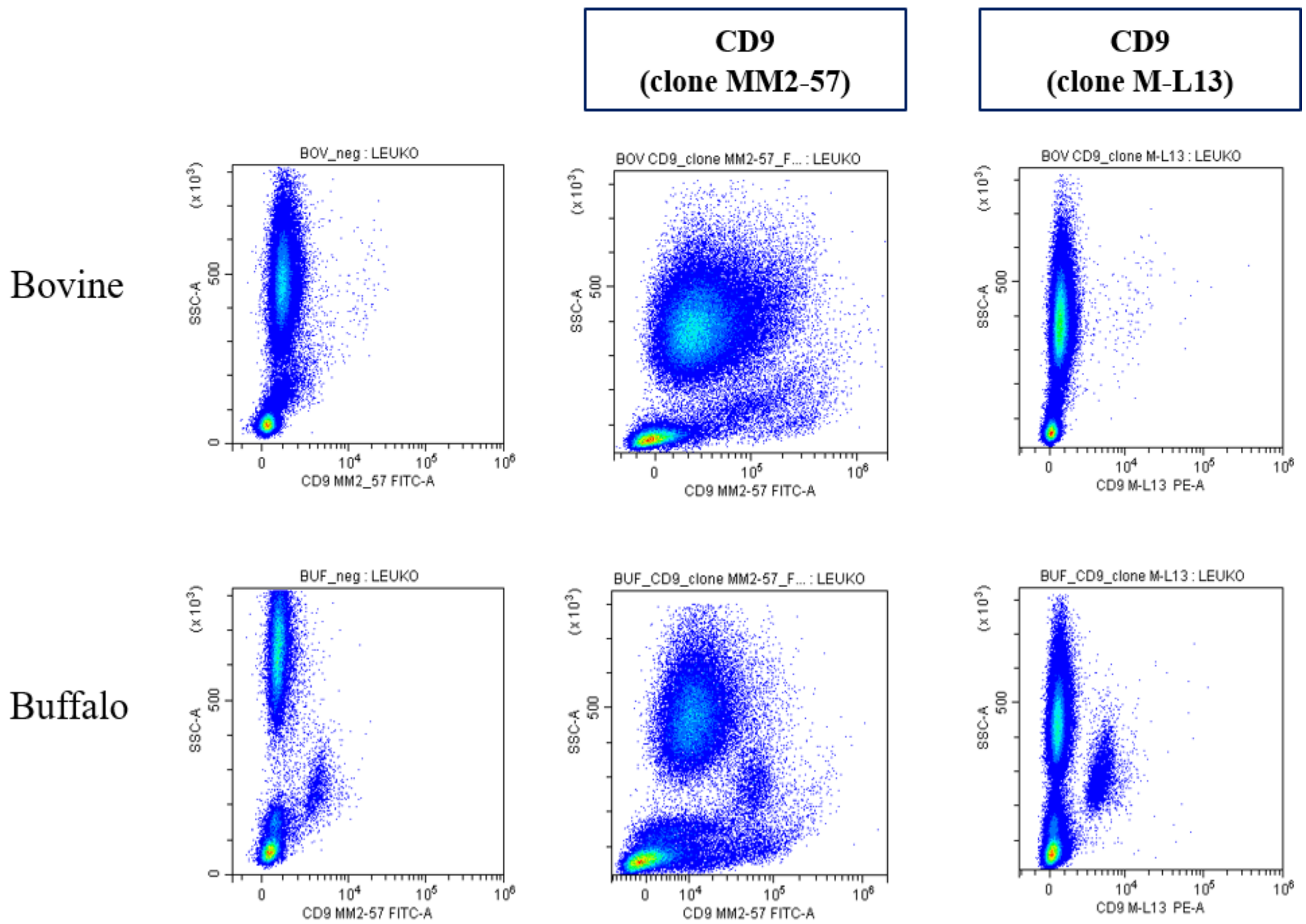

Supplementary Figure 3. Flow cytometric analysis of CD9 expression across different species using clones MM2-57 and M-L13. Representative dot plots show the side scatter (SSC-A) versus fluorescence intensity for leukocytes from bovine and buffalo samples. The columns represent the negative controls (left), staining with the CD9 clone MM2-57 (middle), and staining with the CD9 clone M-L13 (right). Cross-reactivity and gating patterns vary significantly between species and antibody clones.

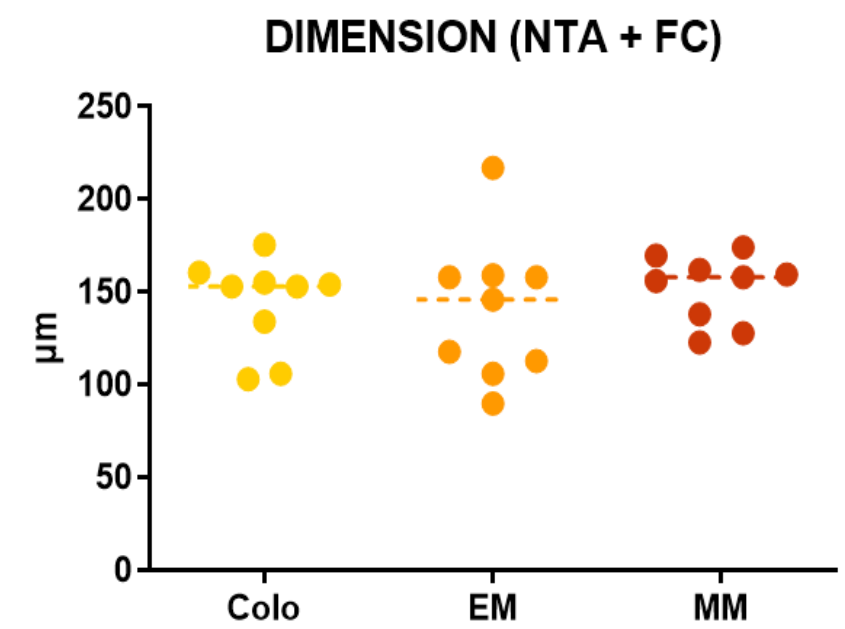

Supplementary Figure 4. Dimension obtained by analysis through NTA and FC.

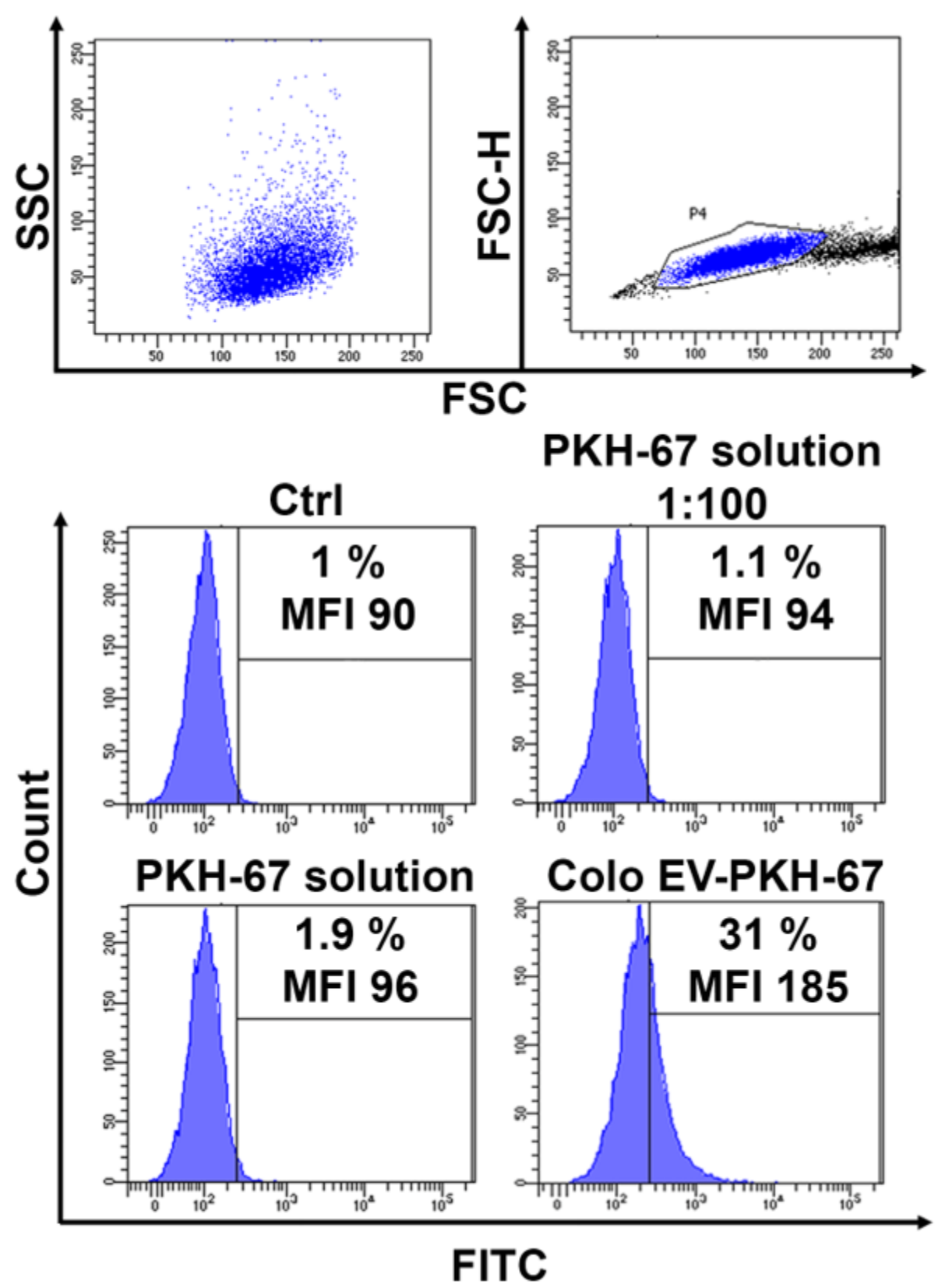

Supplementary Figure 5. Flow cytometric analysis of EV uptake by recipient cells and controls for fluorescent lipophilic dye artifacts. Top panels: Representative physical parameter plots (SSC vs. FSC and FSC-H gating, P4) establishing the cell gating strategy on target recipient cells. Bottom panels: Flow cytometry histograms (FITC channel) evaluating specific PKH67-labeled EV uptake alongside dye-only controls in accordance with MISEV guidelines: Ctrl: Unlabeled control recipient cells establishing baseline autofluorescence. PKH67 solution (1:100 / diluted): Dye-in-buffer/free-dye controls incubated with recipient cells in the absence of EVs, demonstrating negligible background

staining, non-specific binding, or micelle formation. Colo EV-PKH67: Recipient cells incubated with PKH67-labeled Colo EVs, showing a significant shift in fluorescence intensity and percentage of positive cells (31% positive events, MFI 185), confirming true EV uptake.

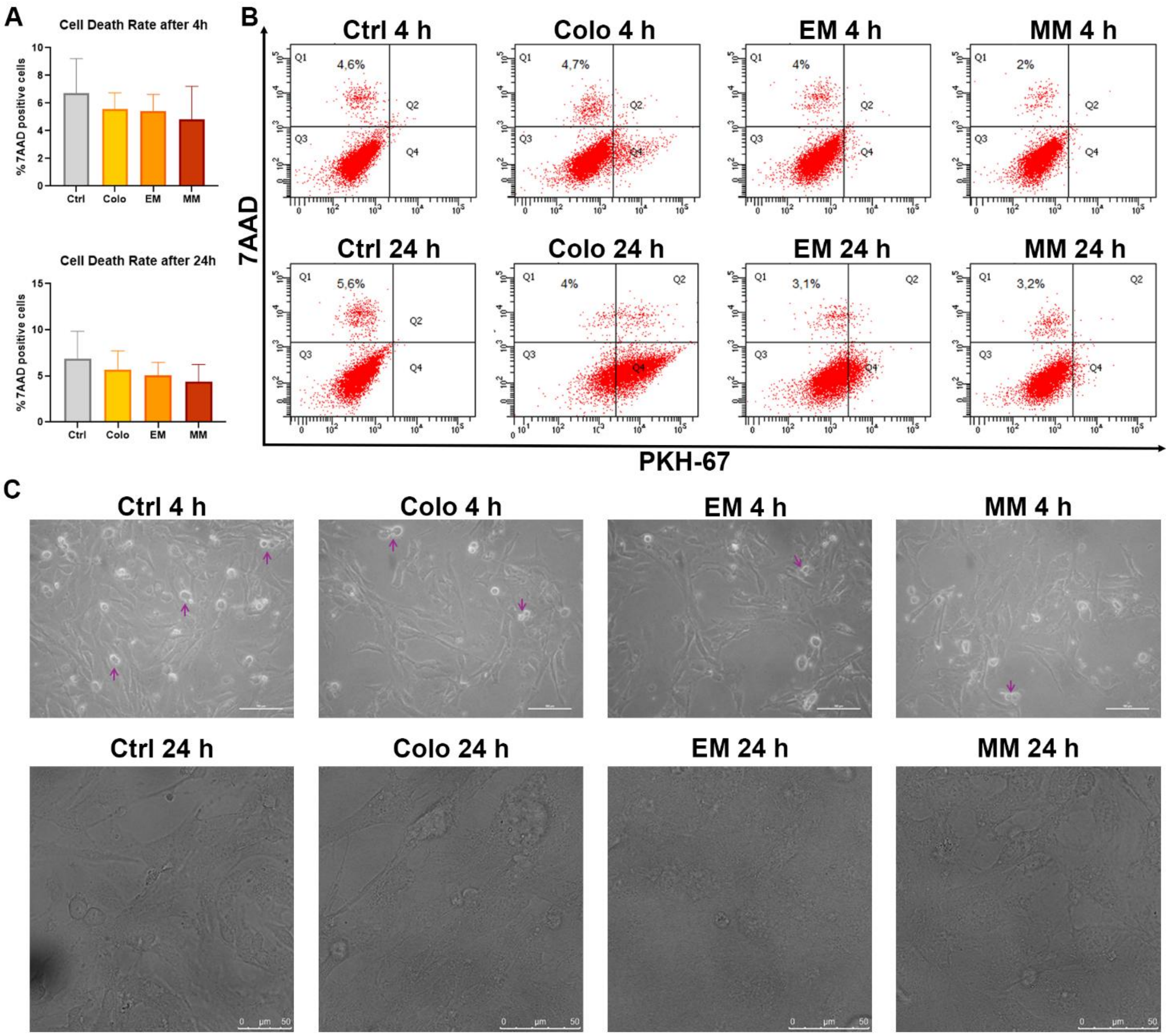

Supplementary Figure 6. Cell viability and morphological analysis of cells treated with mEVs. (A–B) Flow cytometry analysis of cell death rate at 4 h and 24 h post-treatment, evaluated by 7AAD staining. Bar graphs quantify the percentage of 7AAD-positive cells (left), alongside representative dot plots (right) for Control (Ctrl), Colo, EM, and MM conditions. Data are expressed as mean ± SD of n = 3 independent biological replicates. (C) Representative phase-contrast images of cells recorded at 4 h and 24 h post-treatment. Magenta arrows highlight dividing cells. Scale bars: 100 µm for 4 h panels and 50 µm for 24 h panels.

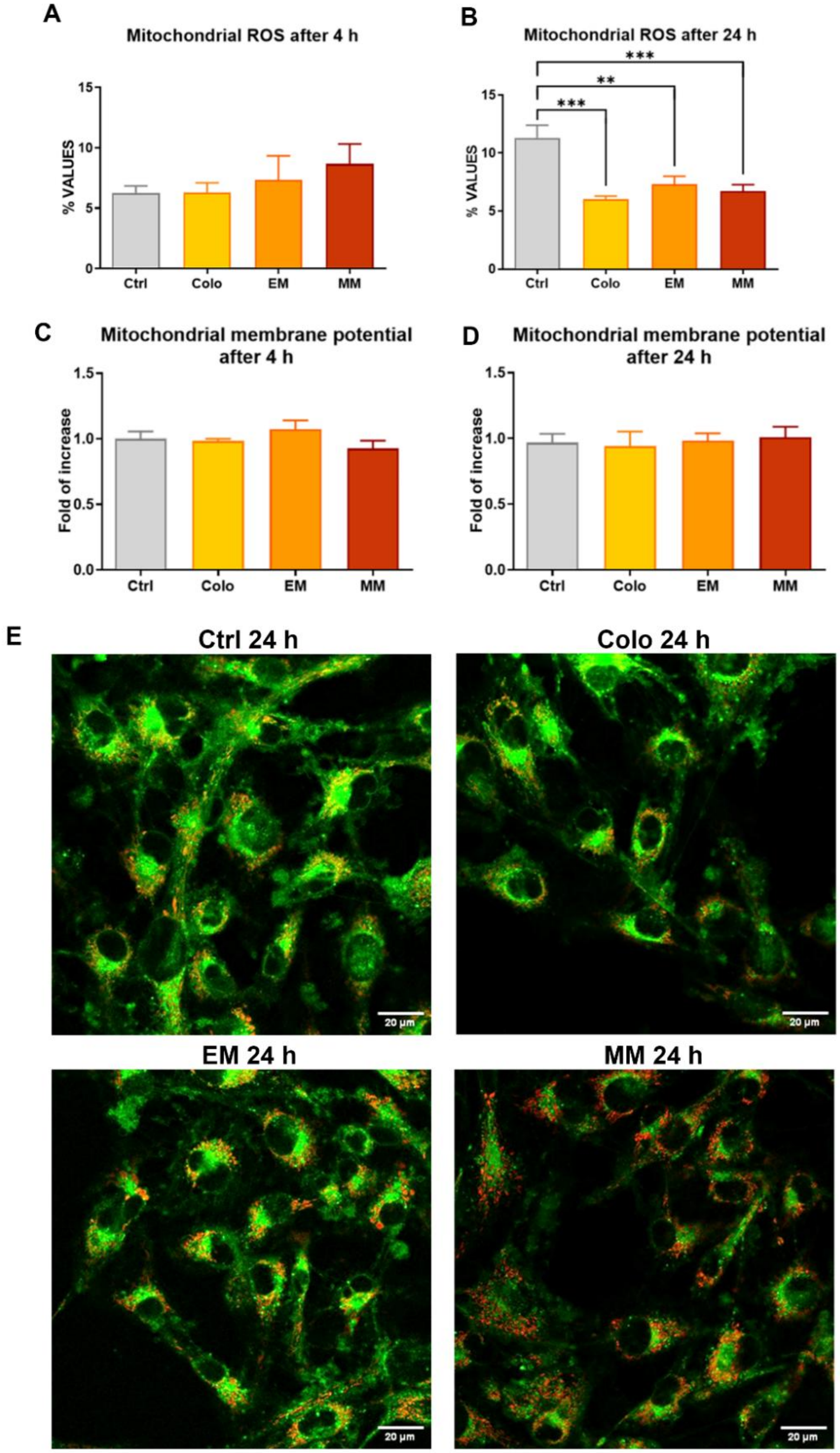

Supplementary Figure 7. A) Mitochondrial ROS production was measured after 4 h of treatment in Ctrl, Colo, EM and MM treated cells. B) Mitochondrial ROS production was measured after 24 h of

treatment. C) Analysis of mitochondrial membrane potential after 4 h of treatment, expressed as fold of increase relative to Ctrl. D) Analysis of mitochondrial membrane potential after 24 h of treatment. E) Representative fluorescence microscopy images showing the localization of lysosomes (green) and mitochondria (red) in Ctrl cells and mEV-treated cells. Data are expressed as mean ± SD of n = 3 independent biological replicates. Statistical significance: **p < 0.01; ***p < 0.001.

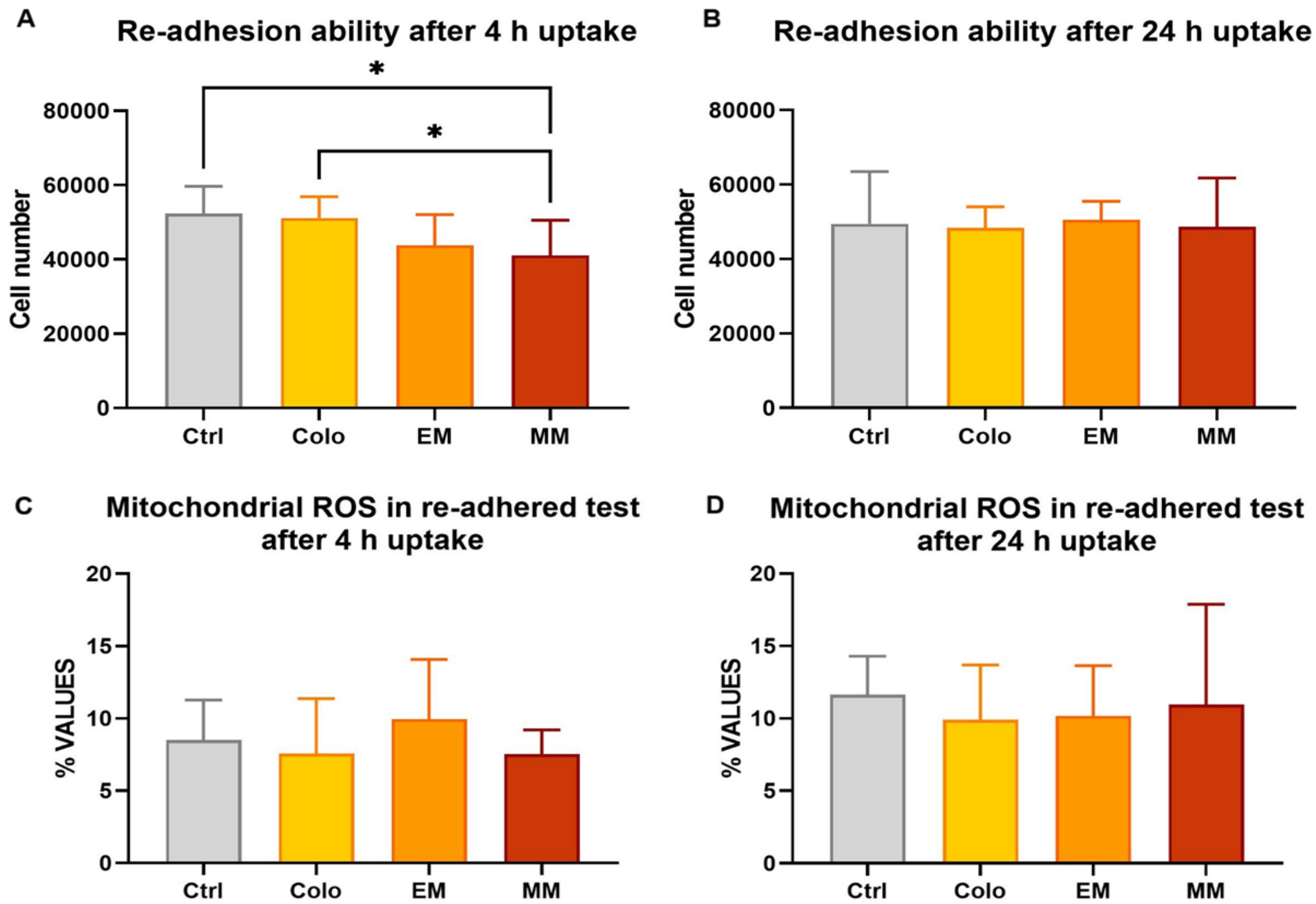

Supplementary Figure 8. Re-adhesion ability and mitochondrial ROS production in BoMac cells. **(A, B)** Evaluation of re-adhesion ability in BoMac cells after an initial 4 h **(A)** and 24 h **(B)** EV uptake. Data are expressed as total cell number. Statistical significance: *p < 0.05. **(C, D)** Mitochondrial ROS production measured in re-adhered BoMac cells after 4 h **(C)** and 24 h **(D)** of initial uptake. Values are reported as percentages.

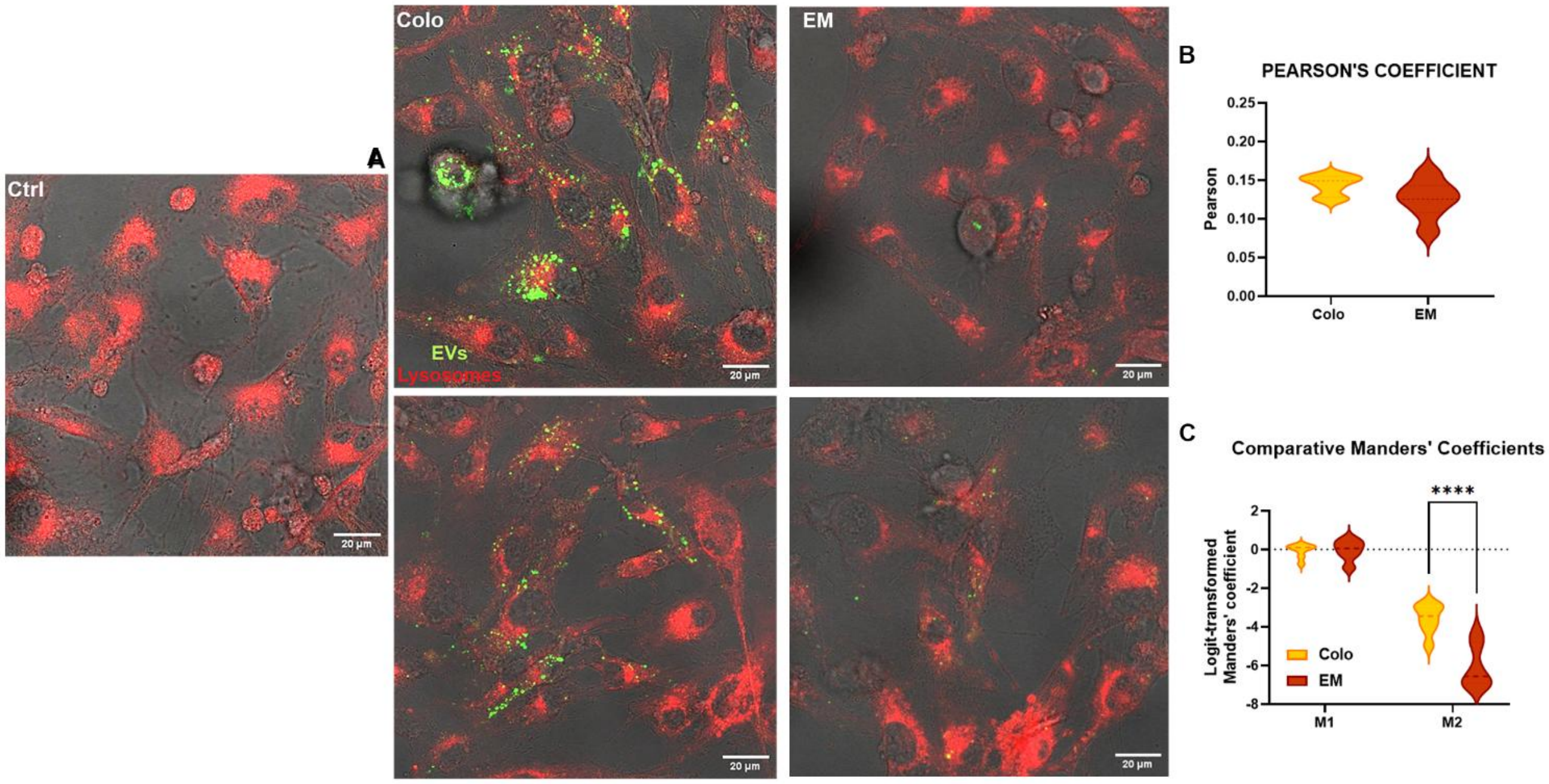

Supplementary Figure 9. Confocal colocalization analysis of EVs with lysosomes. (A) Representative confocal microscopy overlay images (brightfield merged with fluorescence) showing the uptake and subcellular distribution of PKH67 labeled EVs (green) in relation to lysosomes (red) under Control (Ctrl), Colo, and EM treatment conditions. Note: Ctrl and Colo representative images are reproduced from Main Figure 7 to facilitate direct comparative visual assessment with the EM condition. Scale bars = 20 µm. (B) Violin plot quantification of Pearson's correlation coefficient analyzing spatial co-localization between EVs and lysosomes in Colo vs. EM conditions. (C) Violin plots displaying logit-transformed Manders' overlap coefficients (M1: fraction of EVs overlapping with lysosomes; M2: fraction of lysosomes overlapping with EVs). Dashed lines indicate median values; dotted line represents zero co-localization baseline. **** $p < 0.0001$.

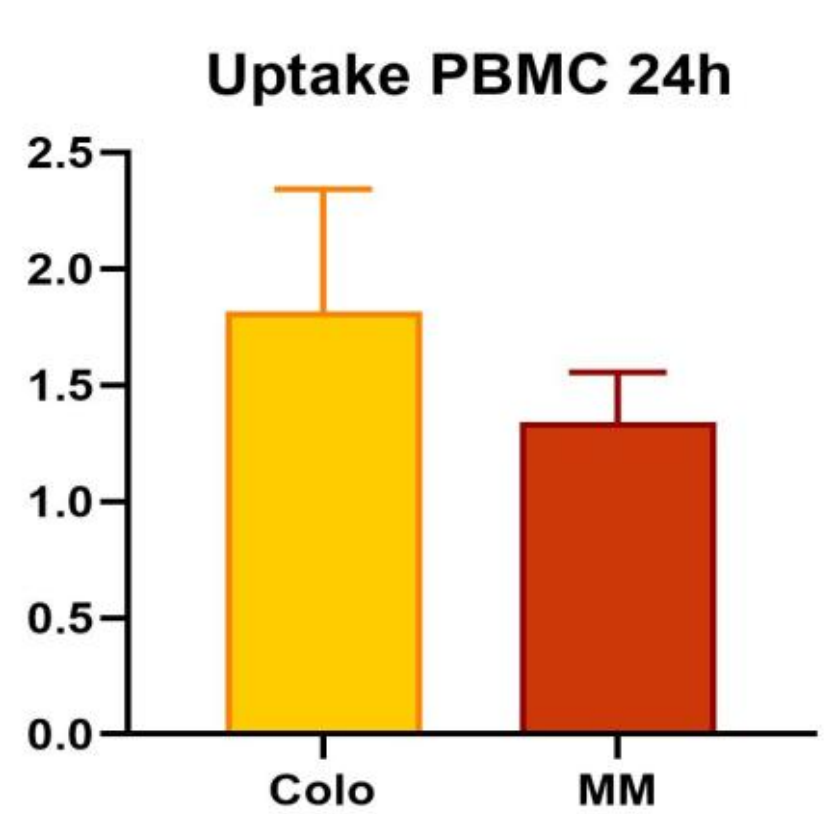

Supplementary Figure 10. EV uptake in PBMC-derived macrophages. Quantitative analysis of EV uptake after 24 h of incubation in PBMC-derived macrophages. Comparison is shown between cells treated with Colo and MM-EVs. Data are expressed as a fold of increase relative to Ctrl. When cells are cryopreserved and subsequently thawed, their endocytic machinery is temporarily compromised, leading to sub-optimal internalization of EVs compared to cells in steady-state culture.
